# β-elemene regulates M1-M2 macrophage balance through the ERK/JNK/P38 MAPK signaling pathway

**DOI:** 10.1038/s42003-022-03369-x

**Published:** 2022-05-31

**Authors:** Yingyu Zhou, Tomohiro Takano, Xuyang Li, Yimei Wang, Rong Wang, Zhangliang Zhu, Masaru Tanokura, Takuya Miyakawa, Satoshi Hachimura

**Affiliations:** 1 Research Center for Food Safety, Graduate School of Agricultural and Life Sciences, The University of Tokyo, Bunkyo-ku, Tokyo 113-8657 Japan; 2 Department of Applied Biological Chemistry, Graduate School of Agricultural and Life Sciences, The University of Tokyo, Bunkyo-ku, Tokyo 113-8657 Japan; 3 Key Laboratory of Industrial Fermentation Microbiology of the Ministry of Education, Tianjin Key Laboratory of Industrial Microbiology, College of Biotechnology, Tianjin University of Science and Technology, National Engineering Laboratory for Industrial Enzymes, Tianjin, 300457 P. R. China

**Keywords:** Obesity, High-throughput screening

## Abstract

Macrophages are classified into classically activated M1 macrophages and alternatively activated M2 macrophages, and the two phenotypes of macrophages are present during the development of various chronic diseases, including obesity-induced inflammation. In the present study, β-elemene, which is contained in various plant substances, is predicted to treat high-fat diet (HFD)-induced macrophage dysfunction based on the Gene Expression Omnibus (GEO) database and experimental validation. β-elemene impacts the imbalance of M1-M2 macrophages by regulating pro-inflammatory cytokines in mouse white adipose tissue both in vitro and in vivo. In addition, the RAW 264 cell line, which are macrophages from mouse ascites, is used to identify the effects of β-elemene on inhibiting bacterial endotoxin lipopolysaccharide (LPS)-induced phosphorylation of mitogen-activated protein kinase (MAPK) pathways. These pathways both induce and are activated by pro-inflammatory cytokines, and they also participate in the process of obesity-induced inflammation. The results highlight that β-elemene may represent a possible macrophage-mediated therapeutic medicine.

## Abstract (web summary)

β-elemene regulates the balance of proinflammatory cytokines in mouse white adipose tissue through MAPK signaling. Targets of β -elemene are predicted based on its 3D structure and include regulators of macrophage-mediated processes.

## Introduction

Microarray technology has been widely applied to identify genetic alterations at the genome level, screen differentially expressed genes (DEGs) and develop novel disease therapies^1^. Along with microarray technology, bioinformatics has been used to predict hub genes of various diseases and related functional pathways^2^. In the current study, Gene Expression Omnibus (GEO) database was applied to clarify the DEGs caused by a high-fat diet (HFD) in a mouse model. As a kind of metabolically triggered inflammation^3^, obesity polarizes various immune cells via the production of inflammatory cytokines^4,5^. Among the immune cell subsets, macrophages play important roles in the processes of forming chronic diseases. Clinical or experimental data have shown that the proportional balance between M1 macrophages (classically activated macrophages) and M2 macrophages (alternatively activated macrophages) is the basis for avoiding autoimmune reactions and chronic inflammatory diseases^6,7^. Although whether a causal relationship exists between inflammation and obesity is still controversial, it is certain that there is a close relationship between inflammation and insulin resistance. When the cells of muscles, fat, or liver cannot respond to insulin, glucose cannot be used to offer energy, insulin resistance occurs. Insulin resistance is the inducer of obesity, high blood pressure, high cholesterol, and type 2 diabetes^8^. In addition, obesity-induced transition of macrophages from M2 polarization to M1 polarization^9,10^ and high expression of pro-inflammatory cytokines^11,12^ will contribute to insulin resistance. Therefore, balancing the two phenotypes of macrophages and alleviating the release of pro-inflammatory cytokines seem to be key points to treating chronic inflammation induced by obesity.

RAW 264 cells, a macrophage cell line derived from mouse ascites, was used to study the mechanisms of potential anti-inflammatory mediators in vitro. When lipopolysaccharide (LPS) is applied to stimulate inflammation in macrophages, the first receptor is Toll-like receptor (TLR) 4, which activates downstream pathways and induces various pro-inflammatory cytokines, such as chemokine (C–C motif) ligand 2 (CCL2), interferon-γ (IFN-γ), interleukin 1β (IL-1β), interleukin 6 (IL-6), and tumor necrosis factor-α (TNFα), as obesity does^13^. These inflammatory mediators induce insulin resistance^14^ and indirectly interfere with insulin signaling pathways by promoting endoplasmic reticulum stress and oxidative stress^15,16^. The mitogen-activated protein kinase (MAPK) pathway mediates cell proliferation and homeostasis, and both are induced and affected by these pro-inflammatory cytokines^17^. MAPK regulates three major pathways, including extracellular signal-regulated kinase (ERK), c-Jun N-terminal kinase (JNK), and p38 MAPK (p38), and can differentially alter the phosphorylation status of numerous proteins, such as transcription factors, signal transduction proteins, cytoskeletal proteins, and some functional enzymes. Studies have shown that in obese mice, knocking out key factors of the MAPK kinase inflammatory pathways can effectively reduce the degree of obesity and insulin resistance^18,19^. Thus, the study of MAPK pathways is becoming a steppingstone for inflammation-mediated diseases, including obesity.

Elemene is a sesquiterpenoid composed of α-elemene, β-elemene, γ-elemene, and δ-elemene^20^. Among them, β-elemene is more effective in anticancer effects than its isomers and can directly kill tumor cells while no effects on other normal cells such as peripheral blood leukocytes at conventional dose^20^. Although strong relationships between inflammation and tumors have been observed, anti-inflammatory function of β-elemene is still poorly understood. Obesity can induce peripheral inflammation and increase the permeability of the gut barrier. The loss of intestinal integrity probably allows for invasion of bacteria and toxins, including the gram-negative bacterium LPS^21^. In contrast, LPS intensifies leaky gut formation and induces adipose tissue-related inflammation, which directly induces insulin resistance and type 2 diabetes^21^. Hence, LPS can mimic obesity-induced inflammation in vitro. The two kinds of biology model will help reveal the effects of β-elemene on inflammation. Based on the chemical structure of β-elemene, the possible targets of β-elemene in regulating macrophage balance in the white adipose tissue of mice were predicted in the present study. To verify this prediction, we then focused on the effects of β-elemene on regulating macrophage balance in the white adipose tissue of mice in vivo and in vitro. In addition, the RAW 264 cell line was used to clarify its effective anti-inflammatory pathways.

## Results

### DEG mining between control-epididymal adipose tissue (EAT) and HFD-EAT at different time courses based on the GEO database

**Fig. 1:**
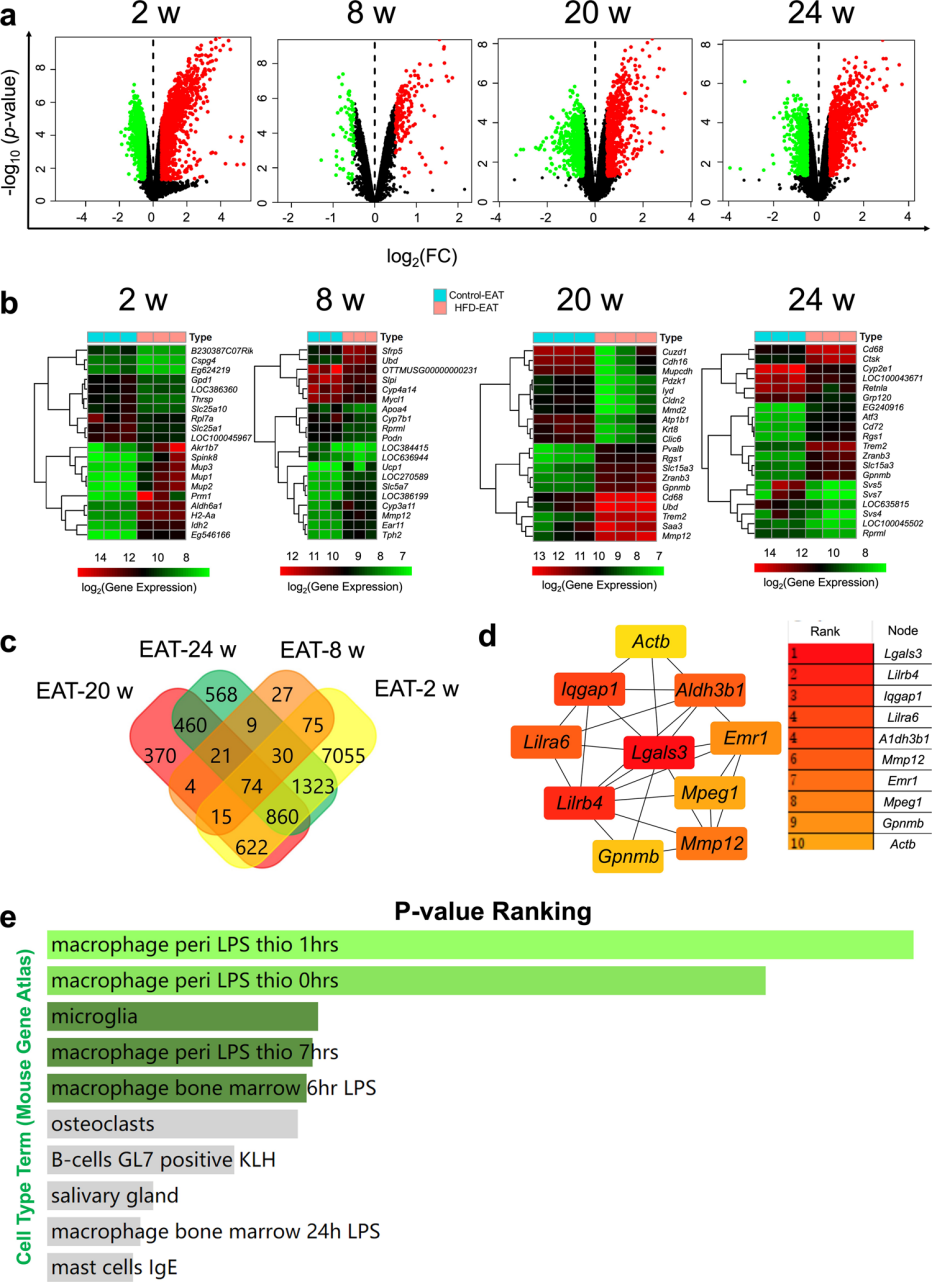
Differential gene mining between control-EAT and HFD-EAT based on the GEO database. **a** Identification of the differentially expressed genes between control-EAT and HFD-EAT at different time courses (2, 8, 20, and 24 weeks). The red and green spots in the HFD group were classified by the upregulated genes and downregulated genes compared with the control, respectively. **b** Heatmap of the top 10 upregulated and top 10 downregulated DEGs in HFD-EAT compared with control-EAT at different time courses (2, 8, 20, and 24 weeks), and the color bar represents differential gene expression magnitude. **c** Venn plot of the differentially expressed genes at different time courses. **d** Top 10 predicted hub genes that could be influenced by the different dietary habits (control or HFD) with high combined scores. (Darker red indicates higher combined scores to be hub genes). **e** Mouse Gene Atlas of overlapping differential genes in the different time courses based on the Enrichr online tool.

Expression profiling by arrays from a previous study (series GSE39549) was selected to help analyze the DEGs between the control-EAT (EAT from mice fed normal diet) and HFD-EAT (EAT from mice fed high-fat diet) groups at different time courses (2, 8, 20, and 24 weeks)^22^. First, the GEO 2 R online tool was used to evaluate the GEO data quantity. The median-centered values (Supplementary Fig. 1a), overlapping density curves (Supplementary Fig. 1b) and straight data lines (Supplementary Fig. 1c) showed that all the samples, including the control-EAT and HFD-EAT groups, had high quantities for the following analyses. In addition, the DEGs between control-EAT and HFD-EAT at different time courses were defined as shown in Fig. 1a. The filters to distinguish the DEGs were *p*-value < 0.05 and |log_2_FC(fold change)| > 0.5 (red and green spots in the HFD-EAT were classified by the upregulated genes and downregulated genes compared with the control-EAT, respectively). Furthermore, a heatmap of the top 10 upregulated and top 10 downregulated DEGs in the HFD-EAT group compared with the control-EAT group at different time courses (2, 8, 20, and 24 weeks) was shown in Fig. 1b. At 2 weeks of HFD, Mup family members, including *Mup1, Mup2*, and *Mup3*, were significantly upregulated in the HFD-EAT group. The Svs family, including *Svs4, Svs5*, and *Svs7*, was significantly downregulated in the HFD-EAT group at 24 weeks of feeding. Moreover, *Mmp12*, which is highly related to immunoreaction^23,24^, was increased by 8 weeks and 20 weeks HFD intake. For different time courses, 74 genes were defined as common differentially expressed genes (Fig. 1c). Among all 74 overlapping genes, the top 10 predicted core genes with high combined scores under the calculation of cytoHubba are shown in Fig. 1d and enriched in immunology-related functions, especially for the functions of macrophages (Fig. 1e) and T cells (Supplementary Fig. 2b). The results highlight that intake of HFD typically influences the immune cells of murine EAT. The Kyoto Encyclopedia of Genes and Genomes (KEGG) pathway analysis and GO functional analysis, including the biological process, molecular function, and cellular component categories of 74 overlapping differential genes in the different time courses, were shown in Supplementary Fig. 2.

### Predicted targets of β-elemene might regulate HFD-induced DEGs

**Fig. 2:**
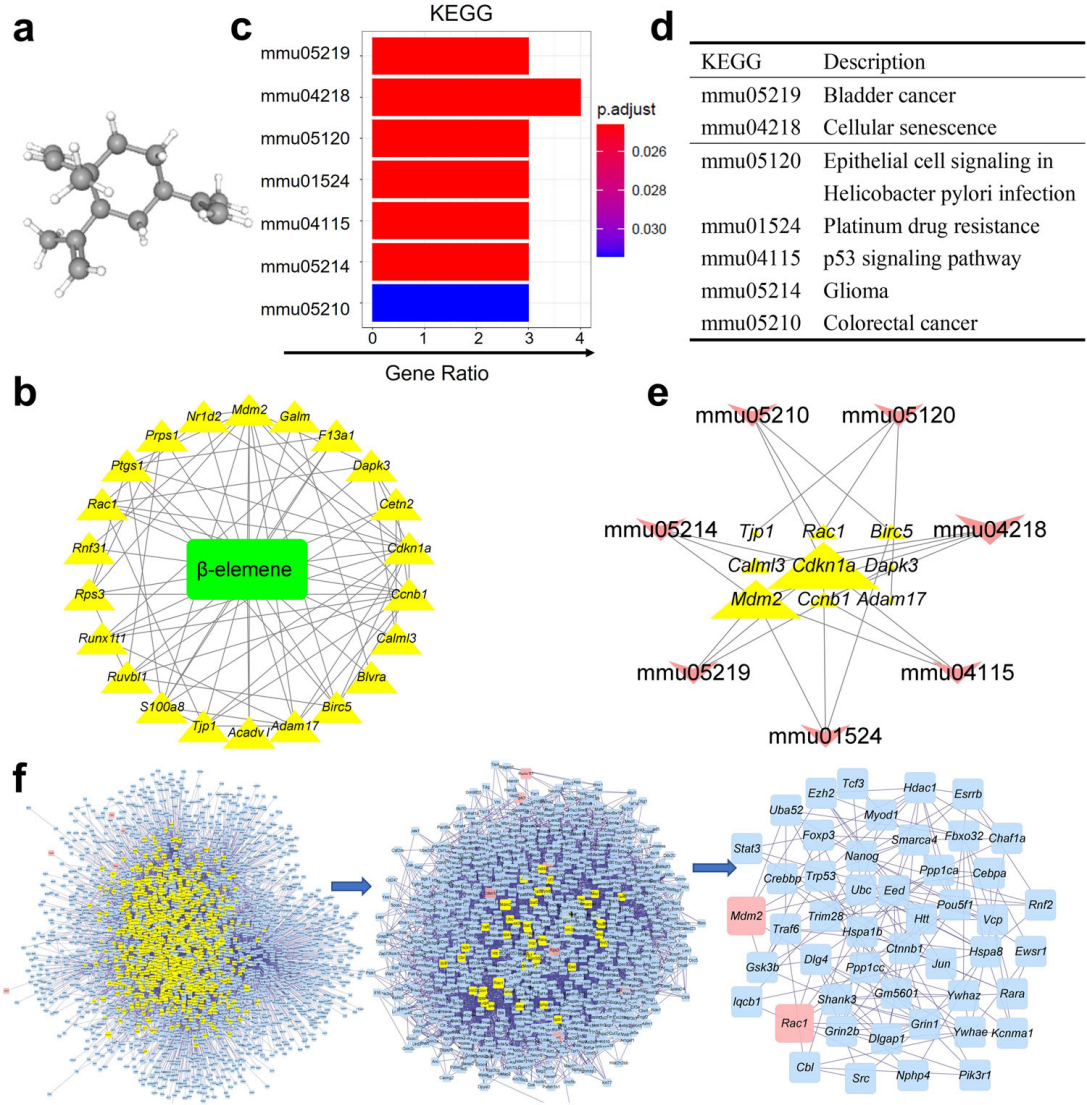
Predicted targets of β-elemene might regulate HFD-induced DEGs. **a** 3D structure of β-elemene. **b** Targets identification of β-elemene on HFD-induced DEGs. **c** Regulated pathway prediction of β-elemene on HFD-induced DEGs (*p*.adjust: *p*-value after correction, Gene Ratio: the proportion of genes enriched in the biological processes). **d** Descriptions of KEGG pathways. **e** Interaction between regulated pathways and targets of β-elemene on HFD-induced DEGs. **f** Network topology of targets of β-elemene on HFD-induced DEGs. A total of 3175 genes (blue) were associated with target DEGs (pink) of β-elemene. Two kinds of filter parameters, degree centrality (DC) and betweenness centrality (BC), were calculated later. After filtering the top 30% DC (the yellow genes shown in the first graph of Fig. 2f), the top 5% BC (the yellow genes shown in the second graph of Fig. 2f) genes were considered the core network.

The anticancer effects of β-elemene are well-known, but its effects on obesity-induced inflammation are poorly understood. Based on the chemical structure of β-elemene is shown in Fig. 2a, the effective targets of β-elemene could be predicted by PharmMapper Server^25^ and TCMSP (Traditional Chinese Medicine Systems Pharmacology Database and Analysis Platform). Because the overlapping 74 DEGs would be the representative genes in HFD-EAT, which are different from normal group, the intersective genes of the 74 DEGs and the predicted effective targets of β-elemene may point to potential therapeutics for treating obesity-induced disease. The effective targets of β-elemene and their interactions are also predicted in Fig. 2b. Based on KEGG pathway and GO analyses (Figs. 2c, 2d, and Supplementary Fig. 3), the targets of β-elemene on HFD-induced DEGs were highly enriched in cancer-related biological processes in agreement with previous reports^26^. In particular, the predicted targets were also enriched in potential macrophage-mediated biological reactions, such as the p53 signaling pathway (mmu04115) and biological process of leukocyte chemotaxis (GO: 0030595). p53 plays an important role in macrophage-initiated inflammatory responses^27^, and chemotactic factors from damaged myofibers could attract macrophages and induce chemotactic responses in myogenic cells^28^. Therefore, the results suggested that β-elemene might involve in the regulation of macrophage functions. In addition, the interaction between the β-elemene target gene and KEGG pathways is shown in Fig. 2e, and the degree of interactions is shown by the size of the delineation (genes are described by triangles, and KEGG pathways are shown by arrows). From Fig. 2e, we can see that cyclin-dependent kinase inhibitor 1 (*Cdn1a*) and murine double minute 2 (*Mdm2*) had higher affinities with KEGGs than other genes. β-elemene target genes were highly enriched at mmu04218 (cellular senescence). Furthermore, based on the *Mus musculus* database, the β-elemene target genes (pink) in Fig. 2b and their neighboring genes (blue) are shown in the first graph of Fig. 2f. Two kinds of filter parameters, degree centrality (DC) and betweenness centrality (BC), were subsequently calculated. After filtering the top 30% DC genes (the yellow genes shown in the first graph of Fig. 2f), the top 5% BC genes (the yellow ones shown in the second graph of Fig. 2f) were considered as the core network. Finally, *Mdm2* and Ras-related C3 botulinum toxin substrate 1 (*Rac1*) appeared to be the core genes in the network topology of predicted targets as shown in the third graph of Fig. 2f. The two genes are reported to regulate macrophage polarization and the insulin signaling pathway^29,30^. This prediction suggested that in addition to its antitumor effects, β-elemene was a potential mediator in regulating macrophage functions. To evaluate this prediction, an obese mice model was used to study the effects of β-elemene on regulating the mRNA expressions of *Mdm2* and *Rac1* in vivo in the Supplementary Fig. 4. We found that mRNA expressions of *Mdm2* and *Rac1* were significantly increased by HFD, and β-elemene could reverse these upregulations.

### β-elemene have no effects on basal activation of fat tissue macrophages

**Fig. 3:**
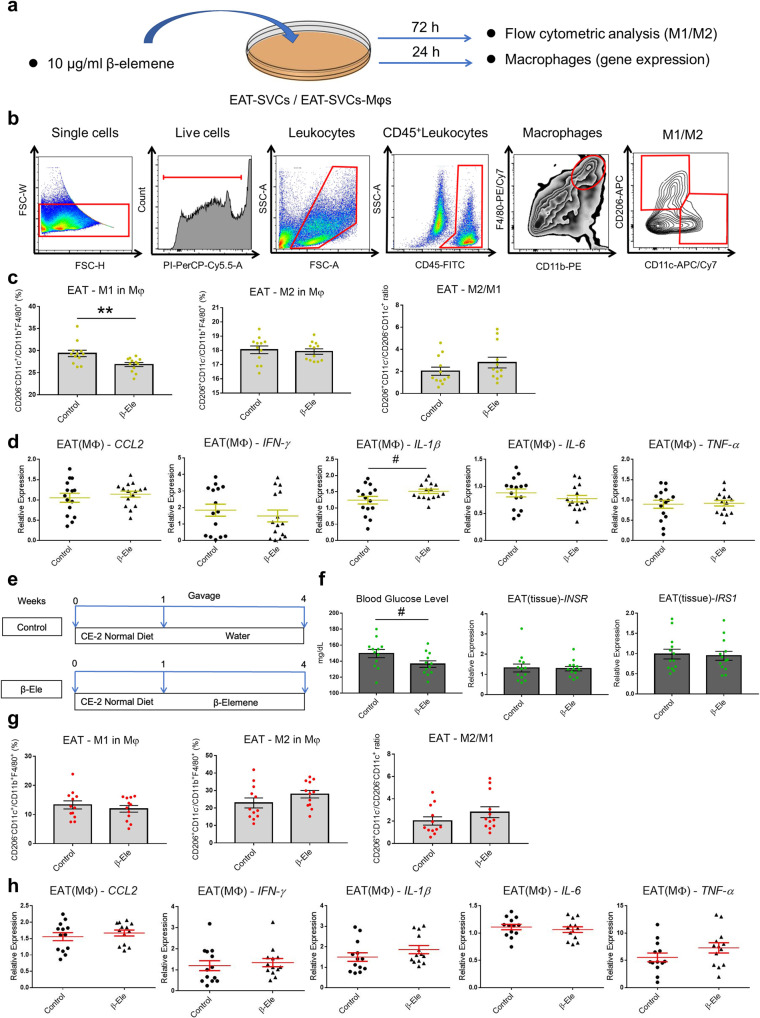
Effects of β-elemene on regulating ATMs in EAT in vitro and in vivo and inflammatory cytokines expressions in normal condition. **a**–**d** Effects of β-elemene on regulating ATMs in EAT and inflammatory cytokines expressions in vitro between control and treatment with β-elemene. Cell culture system of EAT-SVCs in vitro for flow cytometric analysis and RT-PCR (**a**). Analysis process of ATMs of EAT under β-elemene (10 μg/ml) treatment in vitro with flow cytometry (**b**). After culture for 72 h, flow cytometry was used to identify CD206^-^CD11c^+^ M1 Mφs and CD206^+^CD11c^-^ M2 Mφs in total Mφs. Ratio of M1 Mφs in total Mφs, M2 Mφs in total Mφs, and M2 Mφs to M1 Mφs of EAT-SVCs in vitro (**c** *n* = 12). Cytokine gene expressions (*CCL2, IFN-γ, IL-1β, IL-6*, and *TNF-α*) in EAT-SVCs in vitro (**d** *n* = 15). **e**–**h** Effects of β-elemene on regulating ATMs in EAT and inflammatory cytokines expressions in vivo between control and treatment with β-elemene. Schematic presentation of the experimental design for the mouse model (**e**). Effects of β-elemene on mice glucose level (*n* = 12) and insulin resistance related gene expressions (*INSR* and *IRS1*, *n* = 13) (**f**). Ratio of M1 Mφs in Mφs, M2 Mφs in total Mφs, and M2 Mφs to M1 Mφs of EAT-SVCs in vivo (**g** *n* = 12). Cytokine gene expressions (*CCL2, IFN-γ, IL-1β, IL-6*, and *TNF-α*) in EAT-SVCs in vivo (**h** *n* = 13). Control: normal control, β-Ele: SVCs with treatment of β-elemene (10 μg/ml) or β-elemene feeding mice. The results are shown as the mean ± SEM. ^#^*p* < 0.1 and **p < 0.01 versus the control group assessed using two-tailed Student’s *t*-test. The results were from three independent experiments with similar results.

For mice, apart from the four kinds of adipose depots in the abdominal cavity, the paired gonadal and mesenteric depots are important parts composed of white adipose tissue^31^. Inflammatory cytokines directly participate in obesity-related insulin resistance, which indicates a clear link among obesity, diabetes, and chronic inflammation^32^. The stromal vascular cells (SVCs) of white adipose tissue, including EAT and mesenteric adipose tissue (MAT), have the potential to develop adipocytes and are usually applied to study the characteristics of adipose tissue macrophages (ATMs) both in vivo and in vitro^33,34^. SVCs contain preadipocytes, fibroblasts, vascular endothelial cells, and some immune cells, such as ATMs and T cells, all of which show dynamic quantitative and qualitative changes at all times^5,35^. Among these cell subsets, ATMs contribute largely to the homeostasis and integrity of adipocytes^36^. Firstly, the effects of β-elemene on regulating ATMs in EAT in vitro and in vivo as compared with normal were studied. The cell culture system is shown in Fig. 3a and flow cytometric analysis process is shown in Fig. 3b. We can see that β-elemene decreased the M1 macrophages (identified by the CD206^-^ CD11c^+^ phenotype) ratio in total macrophages (identified by F4/80^+^ CD11b^+^ phenotype) of mice EAT, but not the M2 macrophages ratio (identified by the CD206^+^ CD11c^-^ phenotype) in Fig. 3c. Following the analyses of β-elemene’s effects on regulating the polarization of ATMs of murine white adipose tissue, the gene expression of pro-inflammatory cytokines was examined in vitro. Except for IL-1β, other pro-inflammatory cytokines derived from EAT-ATMs including *CCL2, IFN-γ, IL-6*, and *TNF-α* were not influenced by β-elemene treatment (Fig. 3d). For the in vivo experiments, the effects of β-elemene on the ATMs of normal mice were studied with the schedule shown in Fig. 3e. Insulin receptor (INSR) and insulin receptor substrate 1 (IRS1) are two key signaling proteins related to insulin resistance^37^. As shown in Fig. 3f, β-elemene tended to reduce the blood glucose levels of normal mice, but did not significantly alter the mRNA expression of *INSR* and *IRS1*. The results indicated that β-elemene did not influence the insulin resistance of normal mice. In addition, β-elemene could not regulate the macrophages polarization or the expression of pro-inflammatory cytokines in EAT-ATMs after feeding β-elemene to mice (Fig. 3g, h).

### β-elemene regulated the balance between M1 and M2 ATMs in EAT and MAT, modifying inflammation-related cytokines in vitro and in vivo

**Fig. 4:**
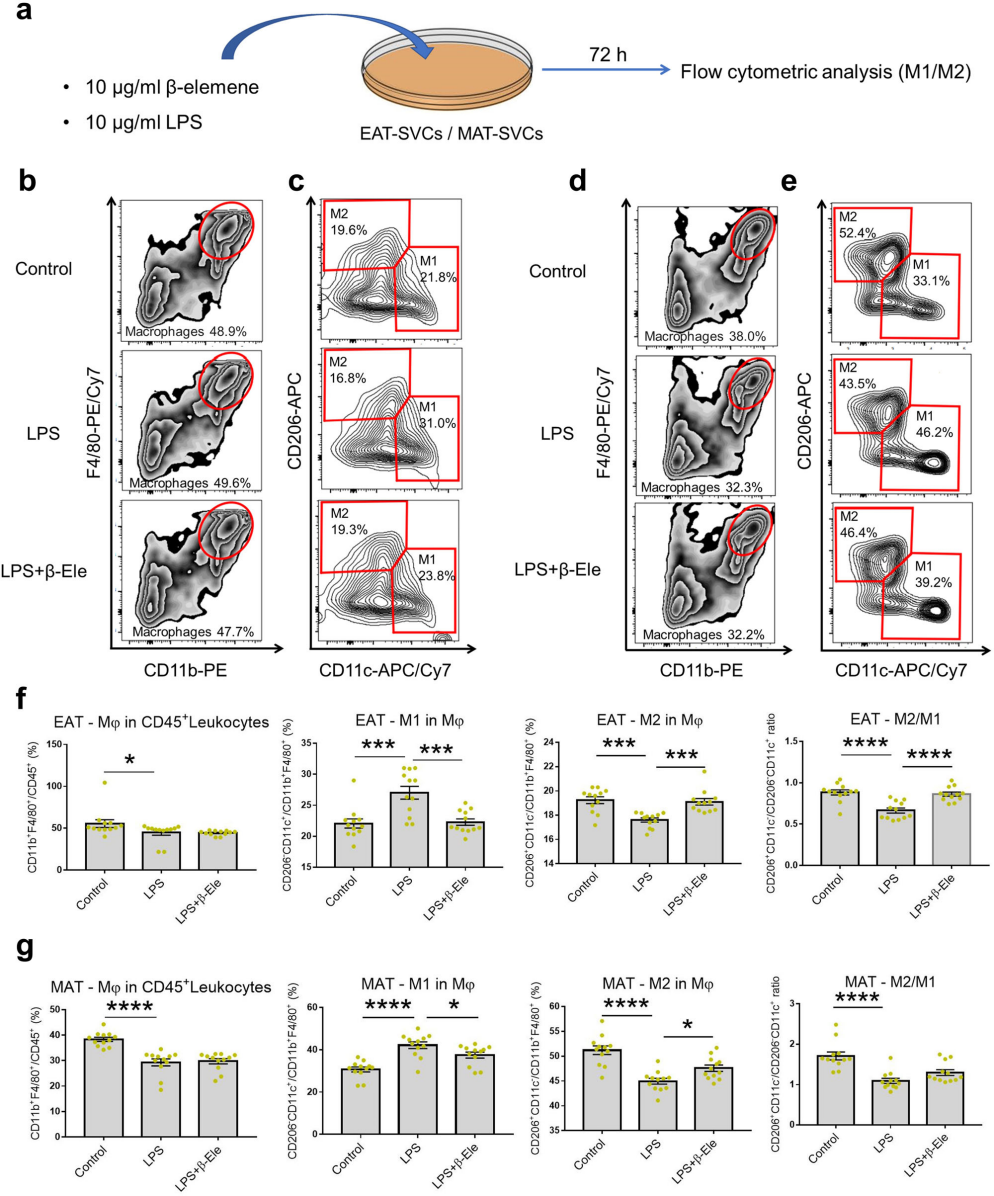
Effects of β-elemene on regulating ATMs in white adipose tissue under LPS stimulation in vitro. **a** Cell culture system of EAT/MAT-SVCs under LPS stimulation in vitro for flow cytometric analysis. **b**–**e** SVCs of EAT or MAT were cultured with LPS (10 μg/ml) and β-elemene (10 μg/ml). After culture for 72 h, flow cytometry was used to identify F4/80^+^CD11b^+^ Mφs in CD45^+^leukocytes, CD206^−^CD11c^+^ M1 Mφs and CD206^+^CD11c^−^ M2 Mφs in total Mφs. Total Mφs (**b**), M1 and M2 Mφs (**c**) in CD45^+^leukocytes of EAT-SVCs. Total Mφs (**d**), M1 and M2 Mφs (**e**) in CD45^+^leukocytes of MAT-SVCs. **f**, **g** Ratio of Mφs in CD45^+^leukocytes, M1 Mφs in total Mφs, and M2 Mφs to M1 Mφs of EAT-SVCs (**f** *n* = 12) and MAT-SVCs (**g** *n* = 12). Data represent individual wells. Control: normal control, LPS: SVCs with LPS (10 μg/ml) stimulation, LPS + β-Ele: SVCs with LPS (10 μg/ml) stimulation under treatment with β-elemene (10 μg/ml). The results are shown as the mean ± SEM. *p < 0.05, ***p < 0.001, ****p < 0.0001 versus the LPS group assessed using one-way ANOVA followed by Dunnett’s multiple comparisons. The results were from three independent experiments with similar results.

**Fig. 5:**
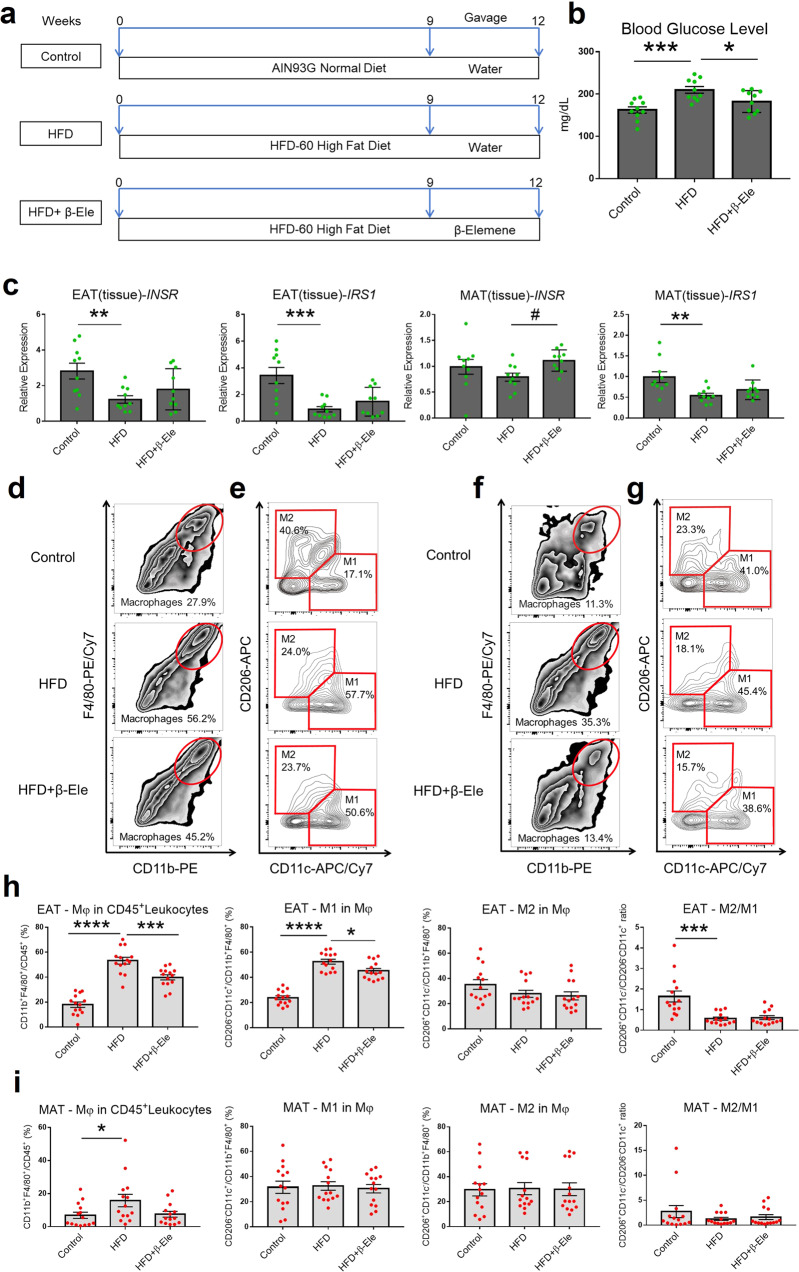
Effects of β-elemene on regulating ATMs of white adipose tissue in obese mice. **a** Schematic presentation of the experimental design for the mouse obesity model. **b**, **c** Effects of β-elemene on mice glucose level (**b** *n* = 10) and insulin resistance related gene expressions (*INSR* and *IRS1*) (**c** *n* = 10). **d**–**g** After obtaining EAT and MAT-SVCs from each mouse, flow cytometry was used to identify F4/80^+^CD11b^+^ Mφs in CD45^+^leukocytes, CD206^−^CD11c^+^ M1 Mφs, and CD206^+^CD11c^−^ M2 Mφs in total Mφs. **h**, **i** Ratio of Mφs in CD45^+^leukocytes, M1 in total Mφs, M2 Mφs in total Mφs, and M2 Mφs to M1 Mφs of EAT-SVCs (**h** *n* = 14) and MAT-SVCs (**i** *n* = 14). Control: normal diet, HFD: high-fat diet, HFD + β-Ele: HFD-induced obese mice under treatment with β-elemene. The results are shown as the mean ± SEM, ^#^*p* < 0.1, *p < 0.05, **p < 0.01, ***p < 0.001, ****p < 0.0001 versus HFD group assessed using one-way ANOVA followed by Dunnett’s multiple comparisons. The results present data from three independent experiments with similar results.

**Fig. 6:**
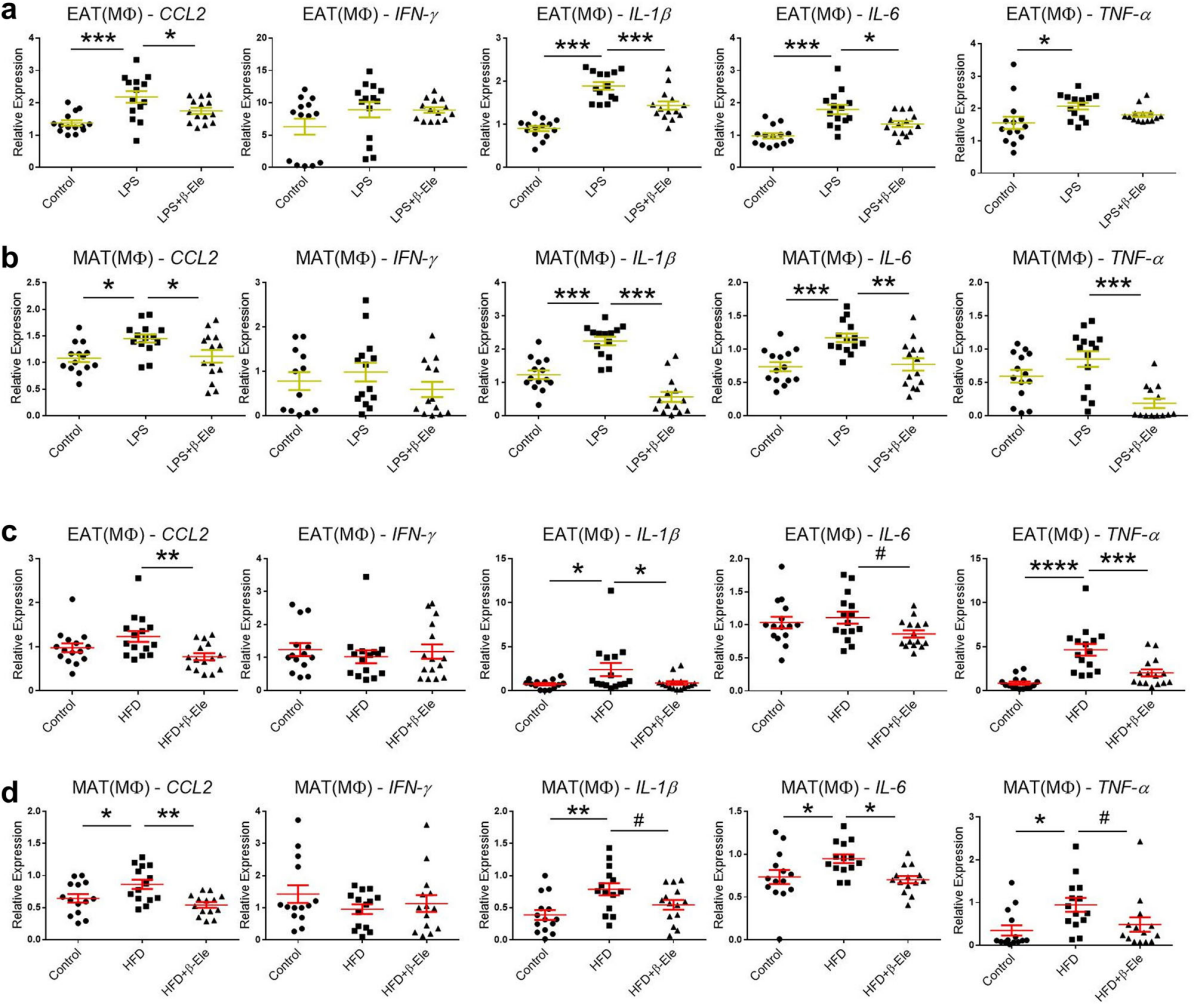
Effects of β-elemene on regulating cytokine gene expression in macrophages of SVCs of white adipose tissue. **a**, **b** Effects of β-elemene on regulating cytokine gene expressions in Mφs of SVCs of white adipose tissue in vitro under LPS stimulation. Control: normal control, LPS, Mφs of SVCs cultured with LPS (10 μg/ml), LPS + β-Ele, Mφs of SVCs cultured with LPS (10 μg/ml) and β-elemene (10 μg/ml). Cytokine gene expressions in Mφs of EAT-SVCs (**a** *n* = 14) and MAT-SVCs (**b** *n* = 14) in vitro, including *CCL2, IFN-γ, IL-1β, IL-6*, and *TNF-α*. Data represent individual wells. The results are shown as the mean ± SEM. *p < 0.05, **p < 0.01, ***p < 0.001 versus LPS group assessed using one-way ANOVA followed by Dunnett’s multiple comparisons. The results were from three independent experiments. **c**, **d** Effects of β-elemene on regulating cytokine gene expressions in Mφs of SVCs of white adipose tissue of obese mice in vivo. Cytokine gene expressions in Mφs of SVCs of EAT (**c** *n* = 15) and MAT (**d** *n* = 14), in**c**luding *CCL2, IFN-γ, IL-1β, IL-6*, and *TNF-α*. Control: normal diet, HFD: high-fat diet, HFD + β-Ele: HFD-induced obese mice under treatment with β-elemene. The results are shown as the mean ± SEM. ^#^*p* < 0.1, *p < 0.05, **p < 0.01, ***p < 0.001, ****p < 0.0001 versus HFD group assessed using one-way ANOVA followed by Dunnett’s multiple comparisons. The results present data from three independent experiments with similar results.

After demonstrating β-elemene’s effects on normal mice, we turned to study the effects of β-elemene on inflammatory conditions in vitro and obesity-induced inflammation in vivo. Following the cell culture for in vitro and flow cytometric analysis shown in Fig. 4a–e (Fig. 4b, c for EAT, Fig. 4d, e for MAT), β-elemene was observed to significantly downregulate M1 macrophages ratio and upregulate M2 macrophages ratio compared with the LPS-induced inflammatory group in the whole population of macrophages for SVC cultures of EAT and MAT (Fig. 4f, g). Similarly, an obese mice model was used to study the effects of β-elemene on treating obesity-induced inflammation in vivo. For the obesity model (Fig. 5a), HFD was shown to increase blood glucose levels significantly and β-elemene could alleviate the phenomenon (Fig. 5b). Moreover, when compared with the control group, lower *INSR* and *IRS1* gene expressions of obese mice have also been observed in Fig. 5c. The results also agreed with previous study^38^. Although there was no significant effect of β-elemene on regulating gene expressions of the two insulin resistance-related proteins of obese mice, an increase tendency could be observed in *INSR* gene expression of obese mice MAT after feeding β-elemene (Fig. 5c). Taken together, β-elemene tended to lower blood glucose levels in normal mice while not influencing the insulin resistance, but decreased blood glucose levels in HFD obese mice, which appeared to be mediated in part by increase in insulin resistance. The effects of β-elemene on regulating macrophages polarization of obese mice were studied next. According to the flow cytometric analysis process (Fig. 5d–g, d, e for EAT, Fig. 5f, g for MAT), the HFD group was observed to have a higher ratio of macrophages to the leukocytes in the EAT-SVCs (Fig. 5h) of EAT of mice compared with the control group, which was also proven by Cho and his colleagues^36^. As shown in Fig. 5h, i, β-elemene downregulated M1 macrophages ratio compared with the HFD group in EAT but did not modify the ratio of M2 macrophages ratio in the whole macrophage population of obese mice. Furthermore, significant effects of β-elemene were observed on downregulating the pro-inflammatory cytokines, including *CCL2, IL-1β, and IL-6* of EAT-Mφs and *CCL2, IL-1β, IL-6*, and *TNF-α* of MAT-Mφs in vitro under LPS stimulation (Fig. 6a, b). Similarly, β-elemene exerted anti-inflammatory effects by significantly decreasing the pro-inflammatory cytokines *CCL2, IL-1β*, and *TNF-α* in EAT-Mφs, *CCL2*, and *IL-6* in MAT-Mφs of obese mice (Fig. 6c, d). These findings showed the anti-inflammatory function of β-elemene in LPS-induced inflammation and obesity mouse models by regulating the balance of ATMs and modifying the pro-inflammatory cytokines in EAT and MAT.

### Effects of β-elemene on LPS-induced changes in the protein levels of inflammation-related cytokines in RAW 264 cells

**Fig. 7:**
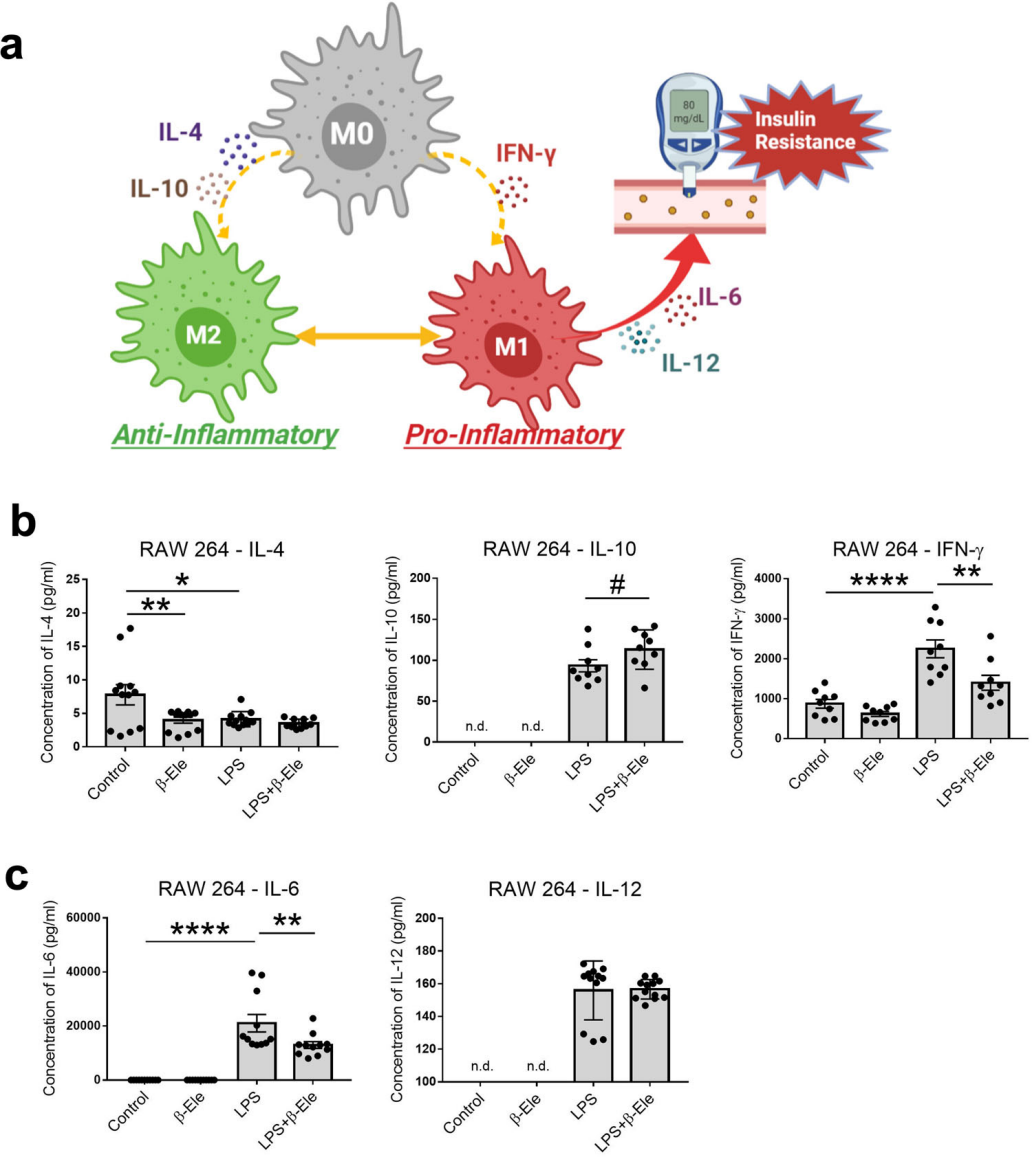
Effects of β-elemene on regulating the protein levels of inflammation-related cytokines in RAW 264 cells. **a** The mechanism of macrophages polarization. **b**, **c** Effects of β-elemene (0 and 10 μg/ml) on LPS (0 and 10 μg/ml)-induced changes in the protein levels of IL-4 (*n* = 12), IL-10 (*n* = 9), and IFN-γ (*n* = 9) (**b**), IL-6 (*n* = 11) and IL-12 (*n* = 12) (**c**) in RAW 264 cells. The results are shown as the mean ± SEM. ^#^*p* < 0.1, *p < 0.05, **p < 0.01, ****p < 0.0001 versus 0 μg/ml LPS or 0 μg/ml β-elemene assessed using one-way ANOVA followed by Tukey’s multiple comparisons or two-tailed Student’s *t*-test. n.d. indicates that the data were below the detection limit. The results present data from three independent experiments.

From the bioinformatics prediction, we found that obesity possibly influences macrophage function, and β-elemene was suggested as a potential macrophage-mediating therapeutic medicine. In the in vitro and in vivo experiments, these suggestions have also been proved to some degree. RAW 264 cells, which are a macrophage cell line, were used to explore the intrinsic mechanisms of β-elemene’s effects on regulating macrophages polarization. LPS would contribute to adipose tissue-related inflammation and is usually used to induce inflammation status^21^. Therefore, we used LPS to study the anti-inflammatory effects of β-elemene in RAW 264 cell system. As shown in Supplementary Fig. 5, even when the concentrations of LPS and β-elemene reached 50 μg/ml, no influence on viability was observed in RAW 264 cells. Thus, the concentrations of LPS (10 μg/ml) and β-elemene (10 μg/ml) were applied in the following experiments. To investigate the alteration of macrophages function after treating β-elemene, production of inflammatory or anti-inflammatory cytokines were measured. M1 macrophages are known to be induced by IFN-γ stimulation, while M2 macrophages are induced by IL-4 and IL-10 ^39^. In addition, M1 macrophages produce cytokines such as IL-6 and IL-12, which contribute to the insulin resistance^40,41^ (Fig. 7a, created with biorender.com). We found that the protein level of anti-inflammatory M2 macrophages inducer IL-4 was obviously decreased by β-elemene treatment as well as LPS stimulation (Fig. 7b). The anti-inflammatory cytokines IL-10 was preferentially increased by β-elemene inference under LPS stimulation (Fig. 7b). Meanwhile, β-elemene significantly decreased the protein levels of IFN-γ, which was increased by LPS stimulation. Moreover, the M1 macrophages marker IL-6 was also obviously reduced by β-elemene treatment, while the IL-12 was not influenced by neither β-elemene or LPS (Fig. 7c).

### β-elemene reduced LPS-induced inflammatory signaling by regulating MAPK pathways

**Fig. 8:**
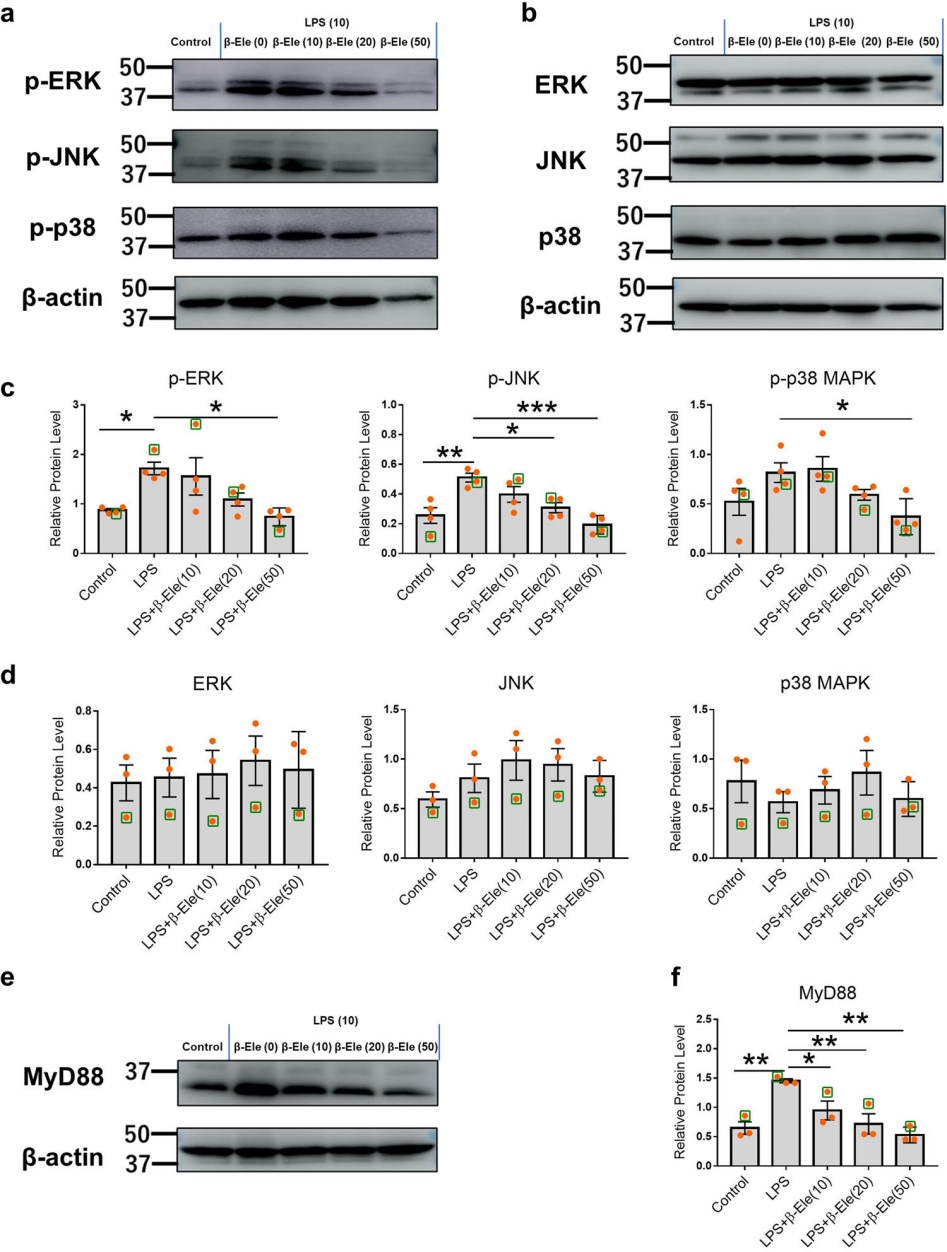
Anti-inflammatory pathways of β-elemene. **a**, **b** Immunoblotting of phosphorylated (**a**) and unphosphorylated (**b**) p42 ERK, p46 JNK, p38 MAPK, and β-actin in RAW 264 cells (β-actin was the internal control). **c** The protein levels of p-ERK, p-JNK, and p-p38 MAPK (*n* = 4). **d** The protein levels of ERK, JNK, and p38 MAPK (*n* = 3). **e** Immunoblotting of MyD88 and β-actin in RAW 264 cells (β-actin was the internal control). **f** Protein level of MyD88 (*n* = 3). LPS: the concentration of LPS was 10 μg/ml in the RAW 264 cell culture system. LPS + β-Ele (0, 10, 20, and 50 μg/ml): LPS (10 μg/ml) and 0, 10, 20, and 50 μg/ml β-elemene were added to the RAW 264 cell culture system. The data of representative figures in **a**, **b**, and **e** were showed in green boxes. The results are shown as the mean ± SEM. *p < 0.05, **p < 0.01, ***p < 0.001 versus LPS group assessed using one-way ANOVA followed by Dunnett’s multiple comparisons. The results represent one of three independent experiments.

The MAPK pathways both are induced and affected by pro-inflammatory cytokines, such as IL-1β, IL-6, IFN-γ, and TNF-α^17^. Therefore, we next studied the effects of β-elemene on regulating MAPK pathways. RAW 264 cells were treated with LPS (10 μg/ml) and β-elemene (0, 10, 20, and 50 μg/ml). After 24 h of culture, the whole-cell protein was subjected to western blotting analysis to examine the effects of β-elemene on alleviating LPS-induced inflammation through regulating MAPK pathways. MAPK pathways can influence various biological processes, such as cell proliferation, stress, inflammation, differentiation, functional synchronization, transformation, and apoptosis. In particular, it could mediate insulin resistance, which seemed to be the key point in treating obesity-induced inflammation^42^. Furthermore, inflammatory cytokines caused by obesity can affect this pathway in turn. Fig. 8a–d shows that with an increased concentration of β-elemene, the levels of the corresponding bands of phosphorylated ERK, JNK, and p38 MAPK decreased, while the levels of unphosphorylated ERK, JNK, and p38 MAPK showed no significant changes, suggesting that β-elemene, especially with high concentrations, regulated LPS-induced MAPK signaling in RAW 264 cells. Furthermore, the expression of myeloid differentiation factor 88 (MyD88) and nuclear factor-kappa B (NF-κB), which is upstream and downstream of the MAPK pathways respectively, were also obviously decreased by β-elemene intervention as shown in Fig. 8e, f and Supplementary Fig. 6. All the original images of western blotting were shown in the Supplementary Fig. 7.

## Discussion

β-elemene was reported to treat various tumors clinically, including lung cancer, liver cancer, brain cancer, bone metastasis, colorectal cancer, etc.^43^. The molecular mechanisms of β-elemene on treating tumor were focused on its effects on inhibiting tumor cell growth and proliferation, modulating immune system, tumor angiogenesis and metastasis, and regulating tumor microenvironment^20^. Liu et al. showed that the pro-inflammatory cytokines, such as TNF-α, IL-1β, and IFN-γ, were enhanced in the arterial vascular wall of obese mice. However, the protein levels of these cytokines in the arteries were reduced remarkably by treating with β-elemene^44^. In addition, β-elemene was also demonstrated to block lipid accumulation and regulate lipid-induced inflammatory pathways with decreasing IL-6 expression in vitro^45^. Although the anti-inflammatory roles of β-elemene have been reported, there are no reports about the effects of β-elemene on regulating obesity-induced macrophages polarization of fat tissue.

Recently, microarray technology has been widely applied to identify genetic alterations at the genome level, screen DEGs, and develop novel cancer therapies^1^. First, the DEGs of HFD-EAT were determined according to the GEO database, and we found that the DEGs were enriched in macrophage-related biological pathways. The results suggested that the HFD induced macrophage dysfunction. In addition, we found that β-elemene possibly affected the regulation of *Mdm2* and *Rac1*. In a previous study, β-elemene induced the high expression of p21-activated protein kinase-interacting protein 1 (PAK1IP1) in gastric cancer cells, and PAK1IP1 inhibited cancer cells proliferation via p53-Mdm2 pathways^46^. Moreover, β-elemene was also suggested to treat sepsis-associated encephalopathy by regulating RAC1/MLK3/p38 signaling pathway^47^. Mdm2 is activated by AKT to degrade p53 and prevent the activation of cell cycle checkpoints downstream of insulin signaling in obesity^29^, while Rac1 has been reported to regulate macrophage morphology^30^ and polarization^48^. In our study, β-elemene was also clarified to decrease the mRNA expressions of *Mdm2* and *Rac1* of macrophages of EAT-SVCs of obese mice, which suggested β-elemene might regulate macrophages polarization of fat tissue through Mdm2- or Rac1-related pathways. The evidence highlights that β-elemene may become a macrophage-mediated therapeutic medicine for curing obesity-induced inflammation. To verify the prediction, experimental validations were also conducted in the present study.

The metabolic and functional states of macrophages can be changed very quickly to adjust to the surrounding microenvironment. It is well known that an imbalance in the phenotypes of macrophages is often associated with chronic disease-induced inflammation^6,7^. Therefore, understanding the status of M1 and M2 macrophages is essential for elucidating the molecular basis of obesity-induced inflammation progression. M1 activation can be evoked by stimulation with TLR ligands, such as LPS-typical gram-negative bacteria in vitro, and is increased in response to IFN-γ derived from Th1 lymphocytes and TNF produced by antigen-presenting cells in vivo^49,50^. In the present study, we found that β-elemene alleviated the M1- polarization caused by LPS in vitro and by obesity in vivo in SVCs of murine white adipose tissue. At the same time, β-elemene downregulated the gene expression of pro-inflammatory cytokines in macrophages, such as *CCL2, IL-1β, IL-6*, and *TNF-α*, which are recognized as induction molecules to switch to M1 polarization^50^.

To explore the intrinsic mechanism of the anti-inflammatory effects of β-elemene, RAW 264 cells, a macrophage cell line derived from mouse ascites, was used to study the anti-inflammatory mechanisms of β-elemene in macrophages. Fang et al. found that pro-inflammatory mediators induced by LPS, such as IL-1β, IL-6, and TNFα, could be significantly suppressed by β-elemene in a dose-dependent manner in a RAW 264.7 cell model^51^. Similar results were also obtained in the present study, and we found that β-elemene had significant effects on regulating the production of inflammatory cytokines at protein levels. RAW 264.7 cells were reported to polarize to M1 macrophage by LPS stimulation^52^. Expressions of IFN-γ and IL-4 was added exogenously to induce M1 and M2 macrophages, respectively^53,54^. In addition, IL-10 was demonstrated to be essential for M2 macrophages polarization^55^. In our study, we found that β-elemene decreased IFN-γ but increased IL-10, which were defined as M1 macrophages inducer and M2 macrophages marker, respectively. Interestingly, β-elemene decreased the protein level of IL-4 in the RAW 264 system as LPS did. The unexpected phenomenon would be explained by the antitumor effects of β-elemene. β-elemene was reported to inhibit pro-tumor M2 phenotypes^53^, and it had no effects on regulating anti-inflammatory M2 macrophages in the obesity animal model according to our results. In addition, as a famous M1 macrophages marker, IL-6 is responsible for the insulin resistance^41^. In current study, we found that β-elemene treatment could significantly decrease LPS- and HFD-induced high expression of *IL-6* both in vivo and in vitro. The discovery corresponded to the effects of β-elemene on inhibiting upregulated blood glucose level of obese mice. These results would provide evidence in explaining the effects of β-elemene on balancing macrophages phenotype of fat tissue of mice in vivo and in vitro with alleviating insulin resistance of obese mice.

Obesity is known to activate the molecules of innate immunity, such as TLRs. In particular, TLR2 and TLR4, which mediate downstream signaling cascades through MyD88, induce the phosphorylation of MAP kinases, including the ERK, JNK, and p38 signaling pathways^56^, and activate inflammatory responses with high production of pro-inflammatory cytokines^57,58^^,^. These cytokines, such as IL-1, IL-6, and TNF, further induce the phosphorylation of JNK and p38^58^. Furthermore, MAP kinases interfere with insulin sensitivity and free fatty acids that have been involved in the development of insulin resistance and type 2 diabetes^59^. These pathways are involved in the formation and deterioration of obesity-induced inflammation. From the results of immunoblotting of LPS-stimulated RAW 264 cells, increasing concentrations of β-elemene corresponded to decreasing bands of p-p42 ERK, p-p46 JNK, and p-p38 MAPK, while unphosphorylated p42 ERK, p46 JNK, and p38 MAPK were stable. In addition, the upstream and downstream pathways of MAPK, MyD88, and NF-κB were also regulated by β-elemene treatment. All these results suggested that β-elemene regulated LPS-induced inflammation in the RAW 264 cell line.

Based on the GEO database, β-elemene was predicted to treat obesity-induced macrophage dysfunction. Experimental validation was applied to identify the effects of β-elemene on regulating the balance between M1 and M2 ATMs in the white adipose tissue of obese mice and inhibiting bacterial endotoxin LPS-induced inflammation by regulating MAPK pathways in mouse macrophages. Although these anatomic structures which are included in the study depend to some extents on subjective interpretation, these results suggested that β-elemene may represent a macrophage-mediated therapeutic medicine.

## Methods

### GEO data mining

Expression profiling by arrays (series GSE39549) revealed differential gene expression between the EAT of normal diet-fed mice and EAT of HFD-fed mice at different time periods (2, 8, 20, and 24 weeks)^22^. The microarray data were from the National Center for Biotechnology Information (NCBI) GEO database. The data quantity, boxplots, expression densities, and moderated *t*-statistic quantile–quantile plot of all the selected samples in GSE39549 were assessed by the GEO 2 R web tool.

### Identification of DEGs

The DEGs between the EAT of control diet-fed mice and HFD-fed mice were screened using R software (version 3.6.3). R software with the R package ‘limma’ from the Bioconductor project was used to calculate |log_2_FC (fold change)| > 0.5 and adj. *p*-values < 0.05, which were considered statistically significant^1^.

### Protein–protein interaction (PPI) construction and hub gene prediction

DEGs were visualized as Venn diagrams by FunRich (version 3.1.3). A PPI network was constructed by STRING to assess functional associations among DEGs^60^. To discover hub genes, the PPI network was analyzed by Cytoscape software (version 3.8.0). The genes with the top 10 combined scores were considered hub genes.

### Prediction of regulated DEGs by the structure of β-elemene

The 3D structure of β-elemene was obtained from PubChem (Compound CID: 6918391). The potential target identification of β-elemene was predicted by the PharmMapper Server and TCMSP using the 3D structure of β-elemene^25^.

### GO and KEGG pathway analysis

KEGG pathway analyses and GO biological process (BP), molecular function (MF), and cellular component (CC) categories of DEGs were predicted by the Enrichr database. The R packages ‘clusterProfiler’, ‘org.Hs.eg.db’, ‘enrichplot’, and ‘DOSE’ from the Bioconductor project were also used to enrich the GO or KEGG pathways of DEGs. All the interactions in the present study were predicted by Cytoscape software (version 3.8.0). The network topology of predicted targets was obtained from applications named BisoGenet and CytoNCA of Cytoscape. Two kinds of filter parameters, degree centrality (DC) and betweenness centrality (BC), were subsequently calculated. After filtering the top 30% DC, the top 5% BC genes were considered the core network.

### Mice

C57BL/6 male mice (8-week-old, weighing 20 ± 3 g) were purchased from Charles River Laboratories, Japan (Yokohama, Japan), and maintained at appropriate temperature (23 ± 2 °C) and humidity (50 ± 5%) with a 12 h light/dark cycle. For in vivo experiments, two kinds of animal experiment models were designed. The former was used to study the effects of β-elemene on the normal mice. The mice were administered with the normal diet (CE-2, CLEA Japan) for 4 weeks, and distilled water or water-dissolved β-elemene (7.5 mg/kg/d, 0.2 ml) was administered by gavage from the second week. The later animal model was used to study the effects of β-elemene on obese mice. The mice were administered with a normal diet (AIN-93G, Oriental Yeast Corporation, Tokyo, Japan) or 60 kcal% fat diet (HFD-60, Oriental Yeast Corporation) separately for 12 weeks. Distilled water or water-dissolved β-elemene (7.5 mg/kg/d, 0.2 ml) was administered by gavage for the last 3 weeks. For the in vitro experiments, all mice were administered the normal diet (CE-2, CLEA Japan) sacrificed at 8–12 weeks of age. Mice blood glucose levels were measured using glucose set (NIPRO, Tokyo, Japan) when they were sacrificed. All the experimental protocols were approved by the Experimental Animal Ethics Committee of the Graduate School of Agricultural and Life Sciences of the University of Tokyo (Approval No. P19-026). All procedures followed the Fundamental Guidelines for Proper Conduct of Animal Experiments and Related Activities in Academic Research Institutions under the jurisdiction of the Ministry of Education, Culture, Sports, Science and Technology, Japan. We have complied with all relevant ethical regulations.

### Reagents and buffers

(−)-β-elemene analytical standard was purchased from Sigma-Aldrich (St. Louis, MO, United States). LPS from Escherichia coli O26 (by phenol extraction) was purchased from FUJIFILM Wako Pure Chemical Corporation (Osaka, Japan).

RPMI media: RPMI 1640 (Nissui Pharmaceutical, Tokyo, Japan) containing 100 U/ml penicillin G potassium (Meiji Seika Pharma, Tokyo, Japan), 100 μg/ml streptomycin sulfate (Meiji Seika Pharma), 50 μM 2-mercaptoethanol (Tokyo Chemical Industry, Tokyo, Japan), 0.03% L-glutamine (FUJIFILM Wako Pure Chemical Corporation), and 0.2% sodium hydrogen carbonate (FUJIFILM Wako Pure Chemical Corporation) was prepared with 10% heat-inactivated FBS (Thermo Fisher Scientific, Waltham, MA, United States). Phosphate-buffered saline (PBS): Dulbecco PBS (-) (4.8 g) (Nissui Pharmaceutical) was dissolved in 500 ml Milli-Q water and sterilized by an autoclave at 121 °C for 20 min. Magnetic-activated cell sorting (MACS) system (Miltenyi Biotec, Bergisch Gladbach, Germany) buffer: Sterilized PBS (-) solution with 0.5% bovine serum albumin (BSA) (FUJIFILM Wako Pure Chemical) and 2.0 mM ethylenediaminetetraacetic acid (EDTA) (FUJIFILM Wako Pure Chemical) was used. Flow cytometry buffer: Sodium azide (FUJIFILM Wako Pure Chemical) was dissolved in PBS (-) solution at a final concentration of 0.1%. After that, the solution was sterilized by an autoclave at 121 °C for 20 min. FBS was added at a final concentration of 1%.

### RAW 264 cell culture and cell viability assay

The RAW 264 cell line was purchased from RIKEN BioResource Research Center Cell Bank (Tsukuba, Ibaraki, Japan). The RAW 264 cell culture medium was composed of 89% Dulbecco’s modified Eagle’s medium (DMEM, Thermo Fisher Scientific), 10% heat-inactivated FBS (Thermo Fisher Scientific), and 1% streptomycin (Nacalai Tesque, Kyoto, Japan). The influences of β-elemene and LPS on RAW 264 cell viability were determined by CCK-8 (Dojindo Molecular Technologies, Kumamoto, Japan). In brief, a density of 3 × 10^4^ RAW 264 cells/well was first seeded into 96-well flat bottom plates (Corning, New York, NY, United States) and cultured for one day until 60–70% of the well area was covered by cells. Different concentrations of β-elemene (0, 1, 5, 10, 20, and 50 μg/ml) and LPS (0, 1, 5, 10, 20, and 50 μg/ml) were applied to each well. After 24 h of incubation, the medium was removed, and the cell viability was measured. A total of 100 μl Cell Counting Kit-8 (CCK-8; Molecular Technologies, Kumamoto, Japan) at a 10-fold dilution was added to each well. Following incubation for 1 h in an incubator (37 °C, 5% CO_2_), the absorbance at 450 nm of each well was measured by a microplate reader (Tecan Trading AG, Männedorf, Switzerland). Finally, the cell viabilities under different treatments were determined by the following formula: % ratio of viable cells = [(*A*_sample_ − *A*_blank_)/(*A*_control_ − *A*_blank_)] × 100%, in which *A*_sample_ is the absorbance of each treated sample, *A*_blank_ is the absorbance of reagent only without cells, and *A*_control_ is the absorbance of the cells in the DMEM culture medium.

### Enzyme-Linked ImmunoSorbent Assay (ELISA)

Purified rabbit antibodies against IL-4, IL-6, IFN-γ, and IL-12 (BD Bioscience, Franklin Lakes, NJ, United States) were diluted 500 times (IL-4, and IL-12) or 1000 times (IL-6 and IFN-γ) in 0.1 M sodium hydrogen phosphate (FUJIFILM Wako Pure Chemical Corporation) in 96-well Nunc Immuno-plates (Thermo Fisher Scientific, 50 μl/well). After storage at 4 °C overnight, the plates were blocked by the addition of 1% BSA/PBS (100 μl/well) at room temperature for 1 h. Samples and standards were diluted with 1% BSA/PBS-Tween 20 (FUJIFILM Wako Pure Chemical Corporation) and incubated for 2 h at room temperature. Biotinylated rabbit antibodies against IL-4 (×2000), IL-6 (×1000), IFN-γ (×2000), and IL-12 (×1000) (BD Bioscience) were diluted with 1% BSA/PBS-Tween (50 μl/well), and the plates were incubated at room temperature for 1 h. Then, streptavidin-alkaline phosphatase (BD Bioscience) was diluted with 1% BSA/PBS-Tween 20 (×5000) and added to plates at 50 μl/well for 30 min at room temperature. 2-Methyl-6-nitroaniline (FUJIFILM Wako Pure Chemical Corporation) was dissolved in diethanolamine buffer (10 mM diethanolamine, 0.5 mM MgCl_2_, pH 9.8, FUJIFILM Wako Pure Chemical Corporation) to a concentration of 1 mg/ml. The solution was added to plates at 50 μl/well, and the plates were incubated in the dark at 37 °C for 30–40 min. The absorbance of all the samples was measured by a microplate reader (Bio-Rad, Hercules, CA, United States) at 405 nm and 490 nm for wavelength correction. The IL-10 concentration was tested by PTEIA ELISA set (BD Biosciences) according to the manufacturer’s instructions.

### Cell preparation and cell culture of adipose tissue

The EAT and MAT of mice were dissociated with 1 mg/ml collagenase type II (Sigma-Aldrich) for 40 min. After filtering with a 114 µm nylon mesh (TOKYO SCREEN, Tokyo, Japan) and centrifuging, the stromal vascular cells (SVCs) were extracted from the cell suspensions after treatment with red blood cell lysis buffer, which is made from ammonium chloride, potassium carbonate, and EDTA (FUJIFILM Wako Pure Chemical Corporation)^61^. The MACS system (Miltenyi Biotec) was used to isolate F4/80^+^ cells using F4/80 MicroBeads Ultrapure (Miltenyi Biotec) from the obtained cells, including EAT-SVCs and MAT-SVCs. The obtained cells were used as macrophages (Mφs).

For the in vivo experiments, EAT-SVCs or MAT-SVCs from each mouse was used for flow cytometric analysis, and EAT-Mφs or MAT-Mφs were for quantitative PCR. For the in vitro experiments, EAT-SVCs (5 × 10^5^cells/well) or MAT-SVCs (5 × 10^5^/well) were seeded into 96-well flat bottom plates (Corning) with LPS (10 μg/ml) and β-elemene (10 μg/ml). After 24 h of culture in an incubator (37 °C, 5% CO_2_), the cells were collected to perform flow cytometric analysis. With the same culture protocol, 5 × 10^4^ EAT-Mφs/well or 5 × 10^4^ MAT-Mφs/well were cultured for quantitative PCR.

### Flow cytometry analysis

Cell staining was performed at 4 °C for 20 min after Fc-blocking (anti-CD16/32 clone 93 antibody, ×200, Biolegend, San Diego, CA, United States) blocking for 15 min with the following monoclonal antibodies: FITC-conjugated anti-CD45 ( × 200, clone: 30-F11) (Biolegend), PE-conjugated anti-CD11b (×200, clone: M1/70) (Biolegend), APC-conjugated anti-CD206 (MMR) (×20, clone: C068C2) (Biolegend), APC/Cy7-conjugated anti-CD11c (×20, clone: MGL1/MGL2) (Biolegend), biotinylated anti-F4/80 (×100, clone: BM8) (Biolegend), and streptavidin PE-Cy7 conjugate (×100) for 20 min. Then the propidium iodide (PI, PerCP/Cy5.5 conjugate, 20 μg/ml) was used to define dead cells. Fluorescent levels were measured by FACS Verse (BD Biosciences) with 200000 cells. All data were analyzed with FlowJo software (BD Bioscience, version 10).

### Quantitative Polymerase Chain Reaction (PCR)

Adipose tissue was homogenized using QIAzol Lysis Reagent (QIAGEN, Hilden, Germany) with Tissue Ruptor II (QIAGEN) and tissue total RNA was extracted using RNeasy Lipid Tissue Mini Kit (QIAGEN) according to the manufacturer’s instructions. EAT-Mφs and MAT-Mφs were performed by quantitative PCR. Total RNA from the cells was isolated using a QIAshredder (QIAGEN, Hilden, Germany) and RNeasy Mini Kit (QIAGEN). cDNA was synthesized from total RNA using SupercSript VILO mastermix (Thermo Fisher Scientific). Quantitative PCR was performed with QuantiTect SYBR Green PCR Kits (QIAGEN) using a CFX Connect Real-Time PCR Detection System (Bio-Rad). All relative gene expression levels were normalized to the gene expression level of glyceraldehyde-3-phosphate dehydrogenase (GAPDH). All the primer sequences for qPCR are shown in Supplementary Table 1.

### Whole protein extracts and immunoblotting of RAW 264 cells

RAW 264 cells (6 × 10^8^) were seeded in 10 cm plates (Corning). After 24 h of culture (60–70% area of plate covered by cells), 10 μg/ml LPS and 10, 20, and 50 μg/ml β-elemene were added to the plates and then cultured in an incubator (5% CO_2_, 37 °C) for another 24 h. The cells were collected by 0.1% trypsin (FUJIFILM Wako Pure Chemical Corporation), which was dissolved in EDTA-PBS(-). Protease and phosphatase inhibitor cocktail (FUJIFILM Wako Pure Chemical Corporation) was dissolved in the tissue protein extraction reagent (Thermo Fisher Scientific), which was used as protein lysis buffer. The purified protein concentration of each sample was determined by the Bradford method. Sodium dodecyl sulfate-polyacrylamide (FUJIFILM Wako Pure Chemical Corporation) gel electrophoresis was used for the separation of all the sample proteins (20 μg for β-actin, p38 MAPK, SAPK/JNK, p44/42 MAPK, phospho-p38 MAPK, phospho-SAPK/JNK, and phospho-p44/42 MAPK (Erk1/2), 60 μg for MyD88 and NF-κB). Acrylamide gel was electrophoretically transferred to polyvinylidene difluoride membrane (Millipore, Burlington, MA, United States). After blocking in 5% BSA/TBST (TBST buffer: 50 mM Tris-HCl, 150 mM NaCl, 30 mM KCl, 1% Tween 20, pH 7.5) (FUJIFILM Wako Pure Chemical Corporation), the membranes were cultured overnight in the primary rabbit antibody (Cell Signaling Technology, Danvers, MA, United States) at 4 °C. The primary antibodies were β-actin, p38 MAPK, SAPK/JNK, p44/42 MAPK, phospho-p38 MAPK, phospho-SAPK/JNK, phospho-p44/42 MAPK (Erk1/2), MyD88, and NF-κB. Then, the membranes were immersed in the bound antibody, which was stabilized in goat anti-rabbit IgG (Thermo Fisher Scientific) for 1 h at room temperature. Immunoreactivity was measured by an Amersham Imager 680 (Cytiva Marlborough, MA, United States) after addressing the sensitivity substrate (Thermo Fisher Scientific) and analyzed by the attached software (GE Healthcare Life Science, Marlborough, MA, United States).

### Statistics and Reproducibility

All the values are given as the mean ± SEM and were analyzed by one-way ANOVA followed by Dunnett’s multiple comparisons, one-way ANOVA followed by Tukey’s multiple comparisons, or two-tailed Student’s *t*-test using GraphPad Prism software (GraphPad Software, Inc., La Jolla, CA, USA). A *p*-value < 0.05 was considered a significant difference. The sample sizes and number of replicates were shown in the figure legends.

### Reporting summary

Further information on research design is available in the Nature Research Reporting Summary linked to this article.

## Supplementary information

Supplementary Information

Description of Additional Supplementary Files

Supplementary Data

Reporting Summary

## Data Availability

Further information and requests for resources and reagents should be directed to and will be fulfilled by the Lead Contact. All the datasets related to bioinformatics analysis can be acquired in GEO database (https://www.ncbi.nlm.nih.gov/geo/). Uncropped blots are shown in Supplementary Fig. 7. All source data underlying the graphs presented in the main figures are provided as Supplementary Data.
