# Supplementary material for: β-elemene regulates M1-M2 macrophage balance through the ERK/JNK/P38 MAPK signaling pathway
Source: Commun Biol. 2022 May 31;5:519. doi: 10.1038/s42003-022-03369-x (PMC9156783; doi:10.1038/s42003-022-03369-x)
Supplement: Supplementary file 1 — Supplementary Information [file 42003_2022_3369_MOESM1_ESM.pdf]

Supplementary Materials

β-elemene regulates M1-M2 macrophage balance through the ERK/JNK/P38 MAPK signaling pathway

Yingyu Zhou, Tomohiro Takano, Xuyang Li, Yimei Wang, Rong Wang, Zhangliang Zhu, Masaru Tanokura, Takuya Miyakawa and Satoshi Hachimura

Table S1. Primer sequences for qPCR (mouse)

Targets	qPCR primers
GAPDH	5-AGGTCGGTGTGAACGGATTTG-3’ (forward)
	5’-GGGGTCGTTGATGGCAACA-3’ (reverse)
IL-6	5’-CTGCAAGAGACTTCCATCCAG-3’ (forward)
	5’-AGTGGTATAGACAGGTCTGTTGG-3’ (reverse)
TNF-α	5’-CAGGCGGTGCCTATGTCTC-3’ (forward)
	5’-CGATCACCCCGAAGTTCAGTAG-3’ (reverse)
IFN-γ	5’-GCCACGGCACAGTCATTGA-3’ (forward)
	5’-TGCTGATGGCCTGATTGTCTT-3’ (reverse)
CCL2	5’-TTAAAAACCTGGATCGGAACCAA-3’ (forward)
	5’-GCATTAGCTTCAGATTTACGGGT-3’ (reverse)
IL-1β	5’-GAAATGCCACCTTTTGACAGTG-3’ (forward)
	5’-TGGATGCTCTCATCAGGACAG-3’ (reverse)
IL-10	5’-CTTACTGACTGGCATGAGGATCA-3’ (forward)
	5’-GCAGCTCTAGGAGCATGTGG-3’ (reverse)
TGF-β1	5’-CCACCTGCAAGACCATCGAC-3’ (forward)
	5’-CTGGCGAGCCTTAGTTTGGAC-3’ (reverse)
Mdm2	5’-TGTCTGTGTCTACCGAGGGTG-3’ (forward)
	5’-TCCAACGGACTTTAACAACTTCA-3’ (reverse)
Rac1	5’-GAGACGGAGCTGTTGGTAAAA-3’ (forward)
	5’-ATAGGCCCAGATTCACTGGTT-3’ (reverse)
INSR	5’-ATGGGCTTCGGGAGAGGAT-3’ (forward)
	5’-GGATGTCCATACCAGGGCAC-3’ (reverse)
IRS1	5’-CGATGGCTTCTCAGACGTG-3’ (forward)
	5’-CAGCCCGCTTGTTGATGTTG-3’ (reverse)

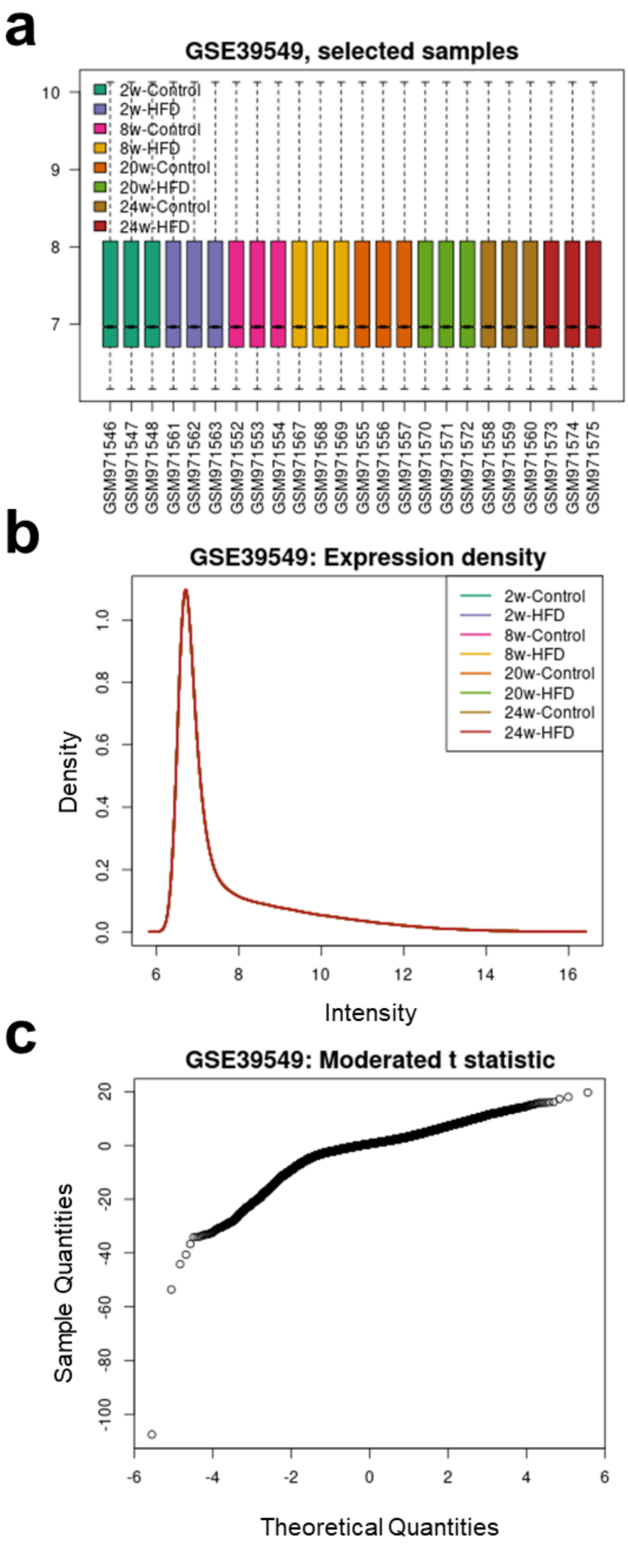

Figure S1. All the samples, including the control-EAT and HFD-EAT samples, had high quantities for the following analyses. a-c Boxplot (**a**), expression densities (**b**), and moderated t-statistic quantile-quantile plot (**c**) of all the selected samples in GSE39549.

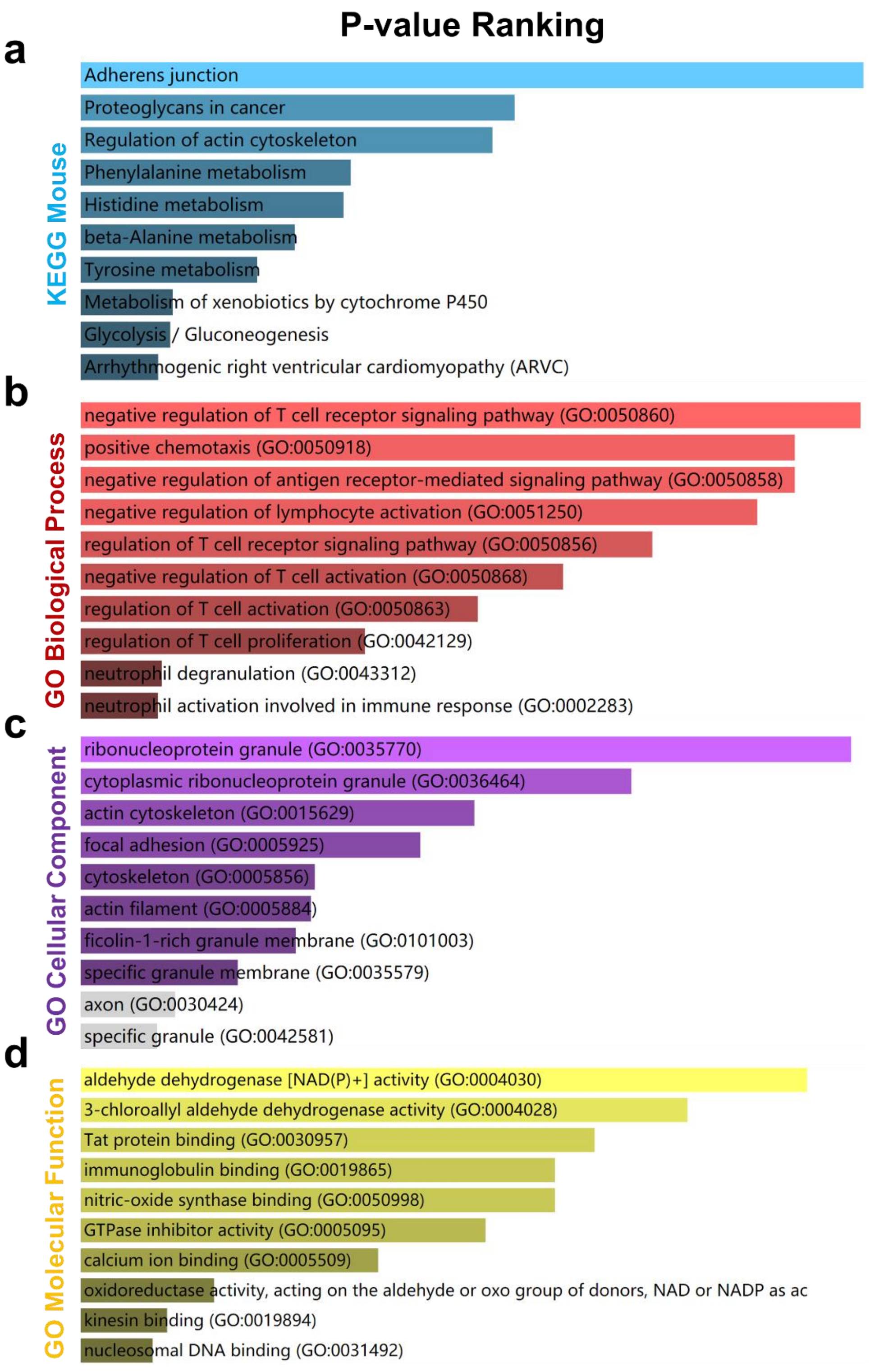

Figure S2. KEGG pathway and GO functional analyses of 74 overlapping differential genes in the different time courses. a-d KEGG pathways (**a**) GO biological process (**b**), GO cellular component (**c**), and GO molecular function (**d**) prediction of overlapping differential genes in the different time courses based on the Enrichr online tool (a smaller *p*-value was described by a longer bar).

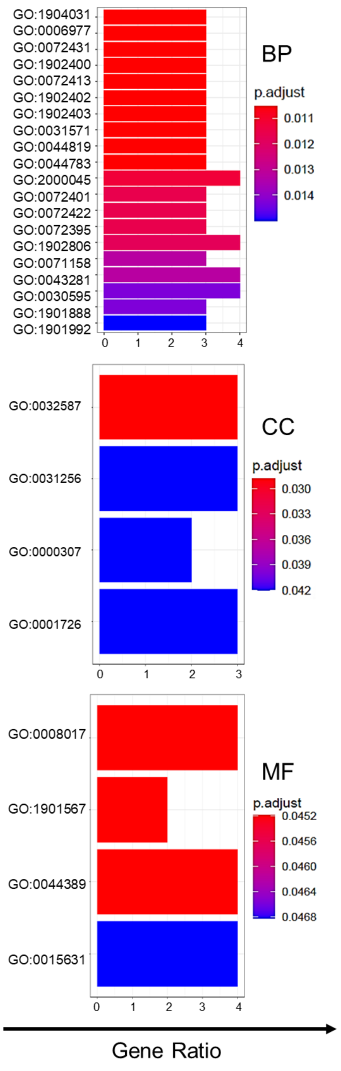

GO ID	Description
GO:1904031	positive regulation of cyclin-dependent protein kinase activity
GO:0006977	DNA damage response, signal transduction by p53 class mediator resulting in cell cycl
GO:0072431	signal transduction involved in mitotic G1 DNA damage checkpoint
GO:1902400	intracellular signal transduction involved in G1 DNA damage checkpoint
GO:0072413	signal transduction involved in mitotic cell cycle checkpoint
GO:1902402	signal transduction involved in mitotic DNA damage checkpoint
GO:1902403	signal transduction involved in mitotic DNA integrity checkpoint
GO:0031571	mitotic G1 DNA damage checkpoint
GO:0044819	mitotic G1/S transition checkpoint
GO:0044783	G1 DNA damage checkpoint
GO:2000045	regulation of G1/S transition of mitotic cell cycle
GO:0072401	signal transduction involved in DNA integrity checkpoint
GO:0072422	signal transduction involved in DNA damage checkpoint
GO:0072395	signal transduction involved in cell cycle checkpoint
GO:1902806	regulation of cell cycle G1/S phase transition
GO:0071158	positive regulation of cell cycle arrest
GO:0043281	regulation of cysteine-type endopeptidase activity involved in apoptotic process
GO:0030595	leukocyte chemotaxis
GO:1901888	regulation of cell junction assembly
GO:1901992	positive regulation of mitotic cell cycle phase transition
GO:0032587	ruffle membrane
GO:0031256	leading edge membrane
GO:0000307	cyclin-dependent protein kinase holoenzyme complex
GO:0001726	ruffle
GO:0008017	microtubule binding
GO:1901567	fatty acid derivative binding
GO:0044389	ubiquitin-like protein ligase binding
GO:0015631	tubulin binding

Figure S3. β-elemene was predicted to treat obesity-induced DEGs. GO biological process (BP), cellular component (CC), and molecular function (MF) prediction of β-elemene on HFD-induced DEGs. (*p*.adjust: p-value after correction, Gene Ratio: the proportion of genes enriched in the biological processes).

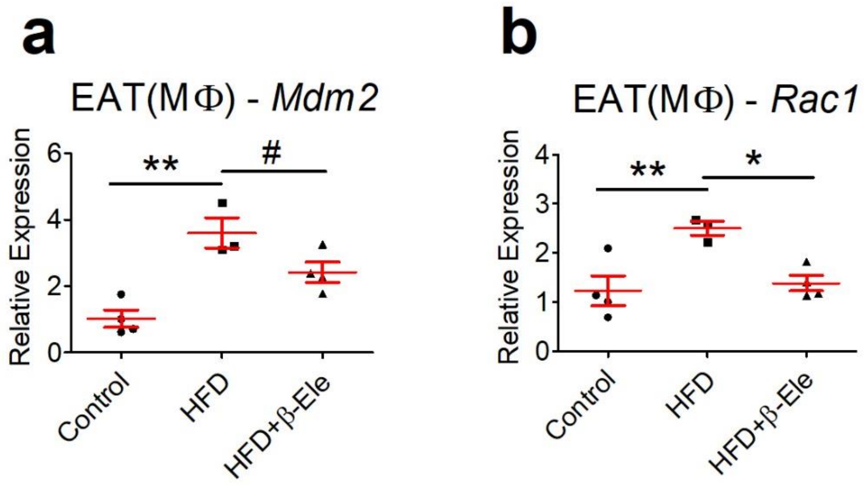

Figure S4. Effects of β-elemene on regulating mRNA expressions of *Mdm2* and *Rac1* in Mφs of SVCs of EAT of obese mice *in vivo*. a, b *Mdm2* mRNA expression (**a**) and *Rac1* mRNA expression (**b**) in Mφs of SVCs of EAT of obese mice. Control (n = 4): normal diet, HFD (n = 3): high-fat diet, HFD + β-Ele (n = 4): HFD-induced obese mice under treatment with β-elemene. The results are shown as the mean ± SEM. $^{\#}p < 0.1$, $^{*}p<0.05$, $^{**}p < 0.01$ versus HFD group assessed using one-way ANOVA followed by Dunnett's multiple comparisons. The results represented one of two independent experiments with similar results.

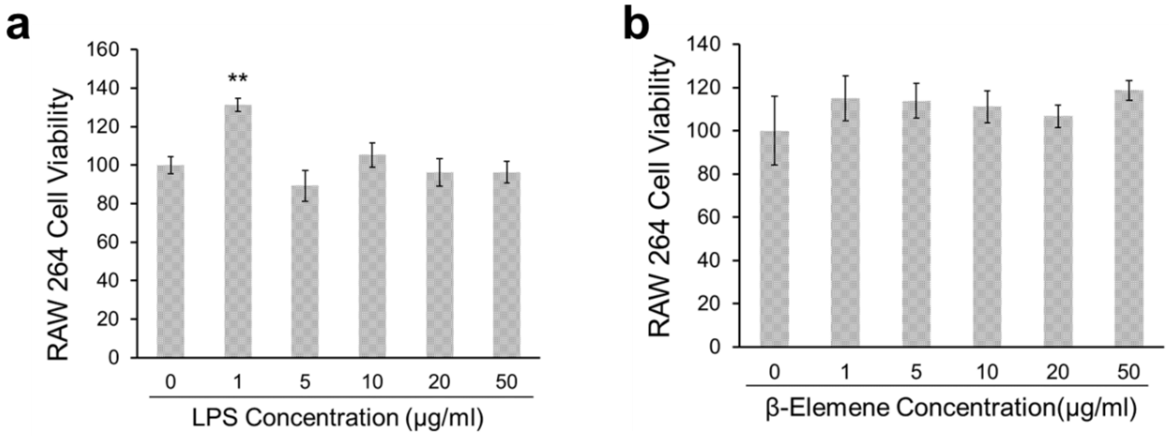

Figure S5. Effects of LPS and β-elemene on RAW 264 cells. a, b Influence of different concentrations of LPS (0, 1, 5, 10, 20, and 50 μg/ml) (**a**) and β-elemene (0, 1, 5, 10, 20 and 50 μg/ml) (**b**) on RAW 264 cell viability under 24 h co-culture. The results are shown as the mean ± SEM (n = 5). $^{**}p < 0.01$ versus 0 μg/ml LPS (**a**) and 0 μg/ml β-elemene (**b**) assessed using one-way ANOVA followed by Dunnett's multiple comparisons.

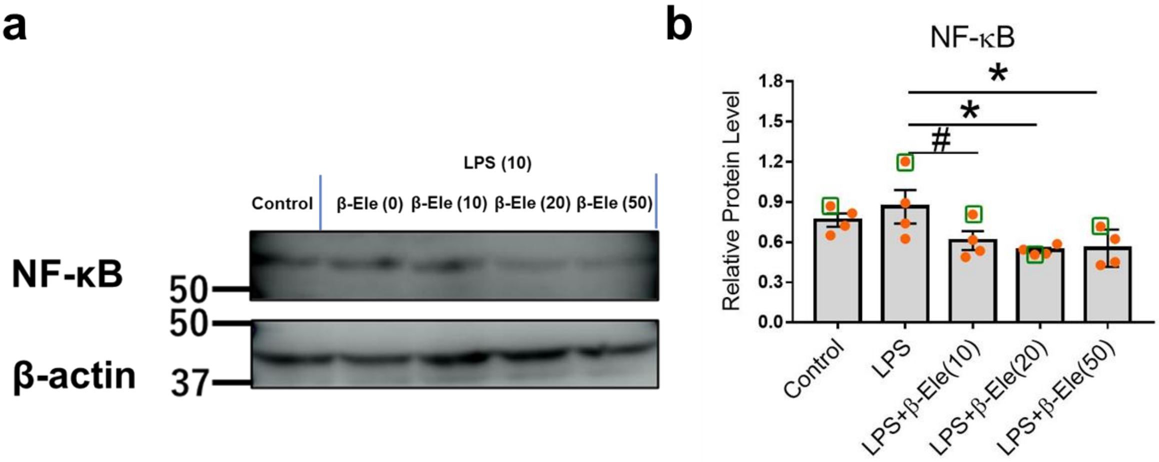

Figure S6. Effects of β-elemene on regulating NF-κB pathway. a Immunoblotting of NF-κB and β-actin in RAW 264 cells (β-actin was the internal control). **b** Protein level of NF-κB and the data of representative figure in **a** were showed in green box. LPS: the concentration of LPS was 10 μg/ml in the RAW 264 cell culture system. β-Ele (0, 10, 20, and 50): LPS (10 μg/ml) and 0, 10, 20, and 50 μg/ml β-elemene were added to the RAW 264 cell culture system. The results are shown as the mean ± SEM (n = 4). $^{\#}p < 0.1$, $^{*}p<0.05$ versus LPS group assessed using one-way ANOVA followed by Dunnett's multiple comparisons.

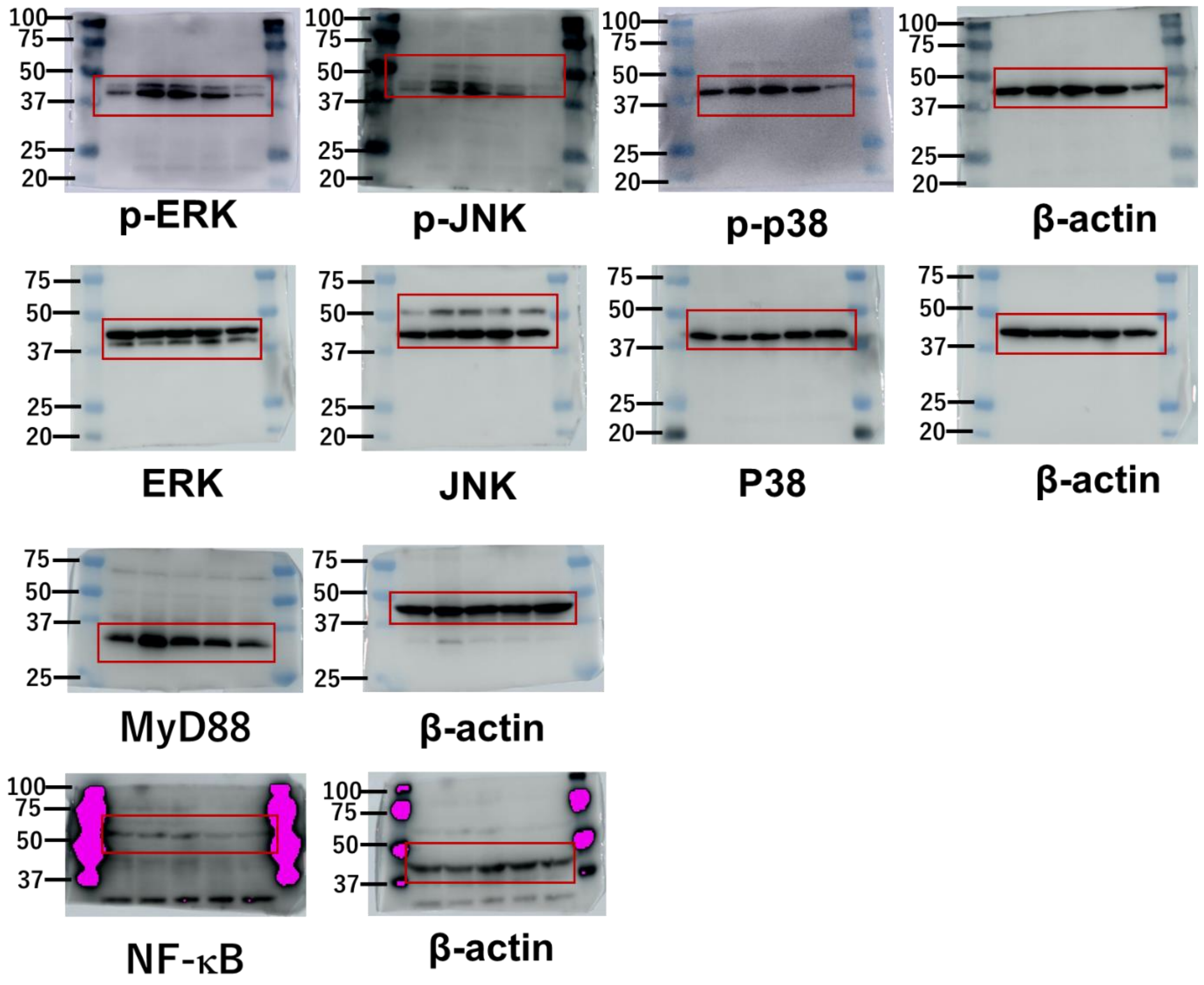

Figure S7. The uncut figures of western blotting in the manuscript.
