# Supplementary material for: β-elemene regulates M1-M2 macrophage balance through the ERK/JNK/P38 MAPK signaling pathway
Source: Commun Biol. 2022 May 31;5:519. doi: 10.1038/s42003-022-03369-x (PMC9156783; doi:10.1038/s42003-022-03369-x)
Supplement: Supplementary file 2 — Description of Additional Supplementary Files [file 42003_2022_3369_MOESM2_ESM.pdf]

Description of Additional Supplementary Files

File name: Supplementary Data
Description: Source data underlying the graphs presented in the main figures.
