# Supplementary material for: β-elemene regulates M1-M2 macrophage balance through the ERK/JNK/P38 MAPK signaling pathway
Source: Commun Biol. 2022 May 31;5:519. doi: 10.1038/s42003-022-03369-x (PMC9156783; doi:10.1038/s42003-022-03369-x)
Supplement: Supplementary file 4 — Reporting Summary [file 42003_2022_3369_MOESM4_ESM.pdf]

nature research

Corresponding author(s): S. Hachimura, T. Miyakawa, M. Tanokura

Last updated by author(s): May 18, 2022

Reporting Summary

Nature Research wishes to improve the reproducibility of the work that we publish. This form provides structure for consistency and transparency in reporting. For further information on Nature Research policies, see our Editorial Policies and the Editorial Policy Checklist.

Statistics

For all statistical analyses, confirm that the following items are present in the figure legend, table legend, main text, or Methods section.

n/a	Confirmed	
☐	☒	The exact sample size (n) for each experimental group/condition, given as a discrete number and unit of measurement
☐	☒	A statement on whether measurements were taken from distinct samples or whether the same sample was measured repeatedly
☐	☒	The statistical test(s) used AND whether they are one- or two-sided<br>*Only common tests should be described solely by name; describe more complex techniques in the Methods section.*
☒	☐	A description of all covariates tested
☐	☒	A description of any assumptions or corrections, such as tests of normality and adjustment for multiple comparisons
☐	☒	A full description of the statistical parameters including central tendency (e.g. means) or other basic estimates (e.g. regression coefficient) AND variation (e.g. standard deviation) or associated estimates of uncertainty (e.g. confidence intervals)
☒	☐	For null hypothesis testing, the test statistic (e.g. F, t, r) with confidence intervals, effect sizes, degrees of freedom and P value noted<br>*Give P values as exact values whenever suitable.*
☒	☐	For Bayesian analysis, information on the choice of priors and Markov chain Monte Carlo settings
☒	☐	For hierarchical and complex designs, identification of the appropriate level for tests and full reporting of outcomes
☒	☐	Estimates of effect sizes (e.g. Cohen's d, Pearson's r), indicating how they were calculated

Our web collection on statistics for biologists contains articles on many of the points above.

Software and code

Policy information about availability of computer code

Data collection	National Center for Biotechnology Information (NCBI) Gene expression omnibus (GEO) database; Enrichr database; FACS Verse (BD Biosciences); CFX Connect Real-Time PCR Detection System (Bio-Rad)
Data analysis	R software (version 3.6.3); FunRich (version 3.1.3); Cytoscape software (version 3.8.0); microplate reader (Bio-Rad); Amersham Imager 680 (Cytiva Marlborough); FlowJo software (version 10); glucose test set

For manuscripts utilizing custom algorithms or software that are central to the research but not yet described in published literature, software must be made available to editors and reviewers. We strongly encourage code deposition in a community repository (e.g. GitHub). See the Nature Research guidelines for submitting code & software for further information.

Data

Policy information about availability of data

All manuscripts must include a data availability statement. This statement should provide the following information, where applicable:
- Accession codes, unique identifiers, or web links for publicly available datasets
- A list of figures that have associated raw data
- A description of any restrictions on data availability

All the Figures, have associated raw data. The data that support the finding of this study are available from the corresponding authors upon reasonable request.

Field-specific reporting

Please select the one below that is the best fit for your research. If you are not sure, read the appropriate sections before making your selection.

☒ Life sciences ☐ Behavioural & social sciences ☐ Ecological, evolutionary & environmental sciences

For a reference copy of the document with all sections, see nature.com/documents/nr-reporting-summary-flat.pdf

Life sciences study design

All studies must disclose on these points even when the disclosure is negative.

Sample size	Sample sizes were determined based on biological variance observed in previous experiments.
Data exclusions	No data were excluded from the analyses.
Replication	n=3-5; Mice experiments and experiments using cells derived from mice, including in vivo in vitro, were each repeated more than 3 times.
Randomization	Randomization was not relevant because there is no allocation of samples/organisms/participants involved in this study.
Blinding	Investigators were not blinded to group allocation during data collection and/or analysis because there is no group allocation involved in this study.

Reporting for specific materials, systems and methods

We require information from authors about some types of materials, experimental systems and methods used in many studies. Here, indicate whether each material, system or method listed is relevant to your study. If you are not sure if a list item applies to your research, read the appropriate section before selecting a response.

Materials & experimental systems

n/a	Involved in the study	
☐	☒	Antibodies
☐	☒	Eukaryotic cell lines
☒	☐	Palaeontology and archaeology
☐	☒	Animals and other organisms
☒	☐	Human research participants
☒	☐	Clinical data
☒	☐	Dual use research of concern

Methods

n/a	Involved in the study	
☒	☐	ChIP-seq
☐	☒	Flow cytometry
☒	☐	MRI-based neuroimaging

Antibodies

Antibodies used	Enzyme-Linked ImmunoSorbent Assay (ELISA): Purified rabbit antibodies against IL-4, IL-6, IL-10, IFN-γ, and IL-12; Biotinylated rabbit antibodies against IL-4, IL-6, IL-10, IFN-γ , and IL-12.<br>Flow cytometry analysis: Anti-CD16/32 (clone 93 anti-body); FITC-conjugated anti-CD45 (clone: 30-F11); PE-conjugated anti-CD11b (clone: M1/70); Alexa Fluor 647 anti- CD206 (MMR) (clone: C068C2); APC/Cy7-conjugated anti-CD11c (clone: MGL1/MGL2); Abiotinylated anti-F4/80 (clone: BM8)
Validation	Purified rabbit antibodies against IL-4 (×500), IL-6 (×1000), IL-10, IFN-γ (×1000), IL-10 (×500), and IL-12 (×500).<br>Biotinylated rabbit antibodies against IL-4 (×2000), IL-6 (×1000), IL-10, IFN-γ (×2000), IL-10 (×1000), and IL-12 (×1000).<br>The dilution ratio of anti-CD16/32 (93) is 1:200 for flow cytometry.<br>The dilution ratio of FITC-conjugated anti-CD45 (30-F11) is 1:200 for flow cytometry.<br>The dilution ratio of PE-conjugated anti-CD11b (M1/70) is 1:200 for flow cytometry.<br>The dilution ratio of Alexa Fluor 647 anti- CD206 (MMR) (C068C2) is 1:20 for flow cytometry.<br>The dilution ratio of APC/Cy7-conjugated anti-CD11c (MGL1/MGL2) is 1:20 for flow cytometry.<br>The dilution ratio of biotinylated anti-F4/80 (BM8) is 1:100 for flow cytometry.<br>The dilution ratio of streptavidin PE-Cy7 conjugate is 1:100 for flow cytometry.<br>The propidium iodide (PI, PerCP/Cy5.5 conjugate, 20 μg/ml) was used to define dead cells for flow cytometry.

Eukaryotic cell lines

Policy information about cell lines

Cell line source(s)	The RAW 264 cell line was purchased from Cell Engineering Division-CELL BANK (Ibaraki, Tsukuba, Japan)

Authentication	Cell lines were authenticated by manufacturer (Cell Engineering Division-CELL BANK)
Mycoplasma contamination	The cells were not tested for Mycoplasma contamination.
Commonly misidentified lines (See ICLAC register)	No commonly misidentified cell lines were used in this study.

Animals and other organisms

Policy information about studies involving animals; ARRIVE guidelines recommended for reporting animal research

Laboratory animals	C57BL/6 male mice (8 w old, weighing 20±3 g) were purchased from Charles River Laboratories, Japan (Yokohama, Japan), and maintained at appropriate temperature (23±2 °C) and humidity (50±5%) with a 12 h light/dark cycle.
Wild animals	N/A
Field-collected samples	N/A
Ethics oversight	All the experimental protocols were approved by the Experimental Animal Ethics Committee of the Graduate School of Agricultural and Life Sciences of the University of Tokyo (Approval No. P19-026). All procedures followed the Fundamental Guidelines for Proper Conduct of Animal Experiments and Related Activities in Academic Research Institutions under the jurisdiction of the Ministry of Education, Culture, Sports, Science and Technology, Japan. We have complied with all relevant ethical regulations.

Note that full information on the approval of the study protocol must also be provided in the manuscript.

Flow Cytometry

Plots

Confirm that:

☒ The axis labels state the marker and fluorochrome used (e.g. CD4-FITC).

☒ The axis scales are clearly visible. Include numbers along axes only for bottom left plot of group (a 'group' is an analysis of identical markers).

☒ All plots are contour plots with outliers or pseudocolor plots.

☒ A numerical value for number of cells or percentage (with statistics) is provided.

Methodology

Sample preparation	Stromal vascular cells of epididymal adipose tissue and mesenteric adipose tissue were used. All the detailed cell preparation and cell culture were provided in the Methods section.
Instrument	FACS Verse (BD Biosciences) was used for data collection.
Software	All data were analyzed with FlowJo (BD Bioscience).
Cell population abundance	200000 cells
Gating strategy	For the Foxp3+CD4+ T cells expression: SSC-H/SSC-W; FSC-H/FSC-W; FSC-A/FSC-H; PI/count; FSC-A/SSC-A; CD45/SSC-A; CD11b/F4/80; CD11c/CD206

☒ Tick this box to confirm that a figure exemplifying the gating strategy is provided in the Supplementary Information.
